# Supplementary material for: Revealing the grammar of small RNA secretion using interpretable machine learning
Source: Cell Genom. 2024 Mar 8;4(4):100522. doi: 10.1016/j.xgen.2024.100522 (PMC11019361; doi:10.1016/j.xgen.2024.100522)
Supplement: Document S1. Figures S1–S5 [file mmc1.pdf]

Cell Genomics, Volume *4*

Supplemental information

Revealing the grammar of small RNA secretion using interpretable machine learning

Bahar Zirak, Mohsen Naghipourfar, Ali Saberi, Delaram Pouyabahar, Amirhossein Zarezadeh, Lixi Luo, Lisa Fish, Doowon Huh, Albertas Navickas, Ali Sharifi-Zarchi, and Hani Goodarzi

Supplementary Figures

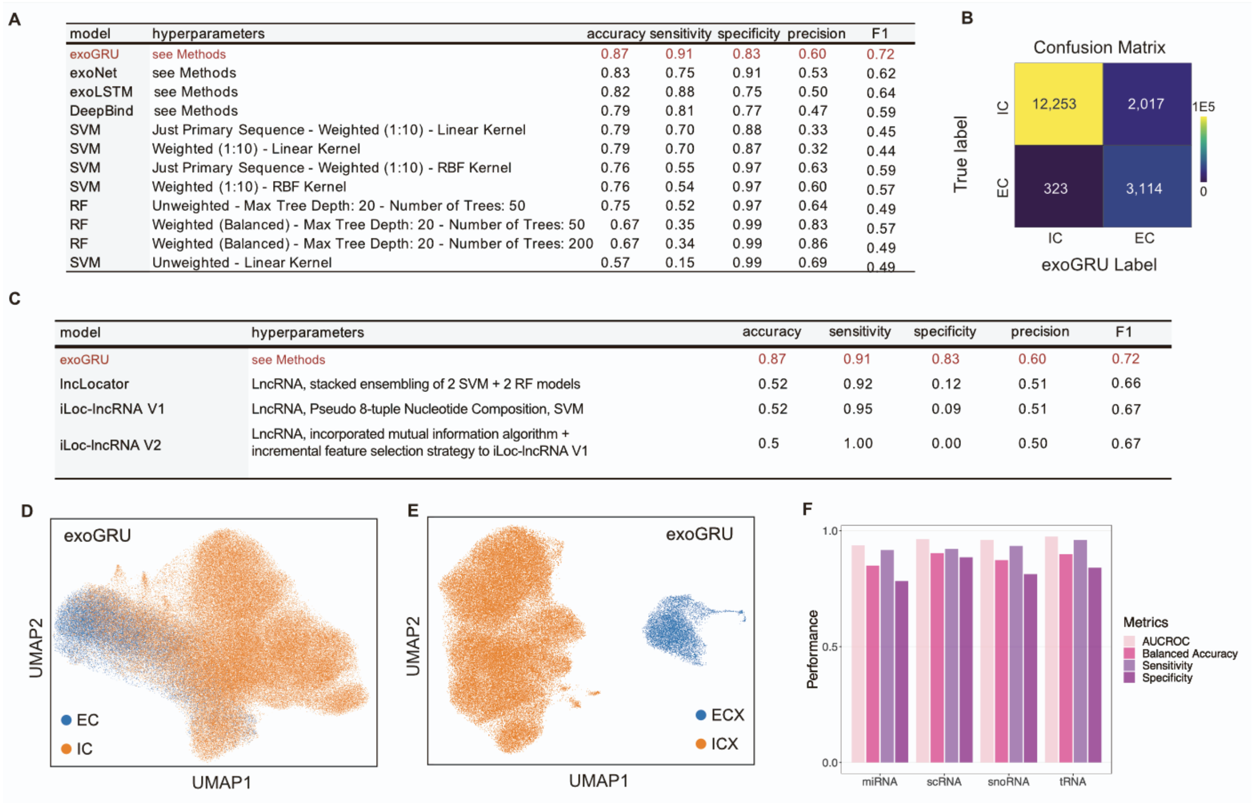

A

model	hyperparameters	accuracy	sensitivity	specificity	precision	F1
exoGRU	see Methods	0.87	0.91	0.83	0.60	0.72
exoNet	see Methods	0.83	0.75	0.91	0.53	0.62
exoLSTM	see Methods	0.82	0.88	0.75	0.50	0.64
DeepBind	see Methods	0.79	0.81	0.77	0.47	0.59
SVM	Just Primary Sequence - Weighted (1:10) - Linear Kernel	0.79	0.70	0.88	0.33	0.45
SVM	Weighted (1:10) - Linear Kernel	0.79	0.70	0.87	0.32	0.44
SVM	Just Primary Sequence - Weighted (1:10) - RBF Kernel	0.76	0.55	0.97	0.63	0.59
SVM	Weighted (1:10) - RBF Kernel	0.76	0.54	0.97	0.60	0.57
RF	Unweighted - Max Tree Depth: 20 - Number of Trees: 50	0.75	0.52	0.97	0.64	0.49
RF	Weighted (Balanced) - Max Tree Depth: 20 - Number of Trees: 50	0.67	0.35	0.99	0.83	0.57
RF	Weighted (Balanced) - Max Tree Depth: 20 - Number of Trees: 200	0.67	0.34	0.99	0.86	0.49
SVM	Unweighted - Linear Kernel	0.57	0.15	0.99	0.69	0.49

C

model	hyperparameters	accuracy	sensitivity	specificity	precision	F1
exoGRU	see Methods	0.87	0.91	0.83	0.60	0.72
IncLocator	LncRNA, stacked ensembling of 2 SVM + 2 RF models	0.52	0.92	0.12	0.51	0.66
iLoc-IncRNA V1	LncRNA, Pseudo 8-tuple Nucleotide Composition, SVM	0.52	0.95	0.09	0.51	0.67
iLoc-IncRNA V2	LncRNA, incorporated mutual information algorithm + incremental feature selection strategy to iLoc-IncRNA V1	0.5	1.00	0.00	0.50	0.67

Supplemental Figure 1. ExoGRU confusion matrix and embedding visualization, related to figure 1
A) Table compares various quality metrics of ExoGRU's performance against different learning models tested**.** In most categories ExoGRU performs better than all the other models. **B)** The confusion matrix illustrates the comparison between ExoGRU-predicted EC and IC labels and their corresponding true labels from the datasets. A total of 3437 sequences with a true EC label were tested, of which approximately 90% were correctly identified as EC by ExoGRU. Similarly, 14270 sequences with a true IC label were tested, with ExoGRU correctly labeling approximately 86% of them as EC. **C)** Comparison of ExoGRU model with other existing models for prediction of RNA subcellular localization. Quality metrics are also listed for ExoGRU and all other existing models **D)** UMAP projection was used to visualize the 64-dimensional embedding of EC vs IC. EC labeled sequences are those with > 0.5 secretion probability and IC sequences have < 0.5 secretion probability as predicted by ExoGRU. **E)** UMAP projection shows the 64-dimensional embedding of high confidence ECX and ICX calls. ECX labeled sequences are those with > 0.95 secretion probability and ICX sequences are those with < 0.05 secretion probability as predicted by ExoGRU. **F)** Comprehensive analysis of ExoGRU's performance in predicting subcellular localization across various small RNA subtypes including miRNA, scRNA, snoRNA, tRNA.

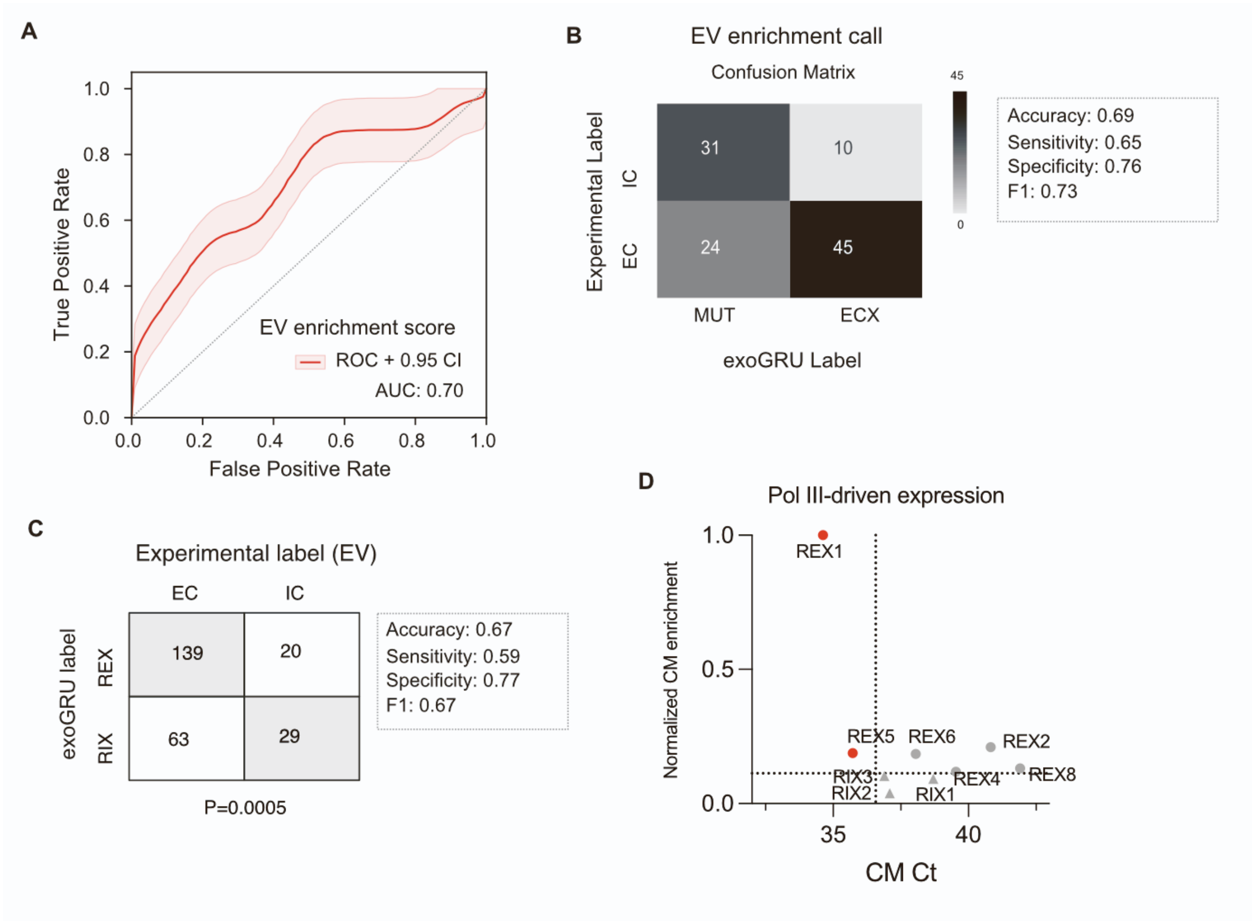

Supplemental Figure 2. ROC curve and confusion matrix for ExoGRU predictions and its experimental validations, related to figure 2. A) ROC curve generated using ECX and MUT experimental EV enrichment score and ExoGRU's localization predictions to measure the association between the experimental vs ExoGRU labels at every classification threshold. The smoothened ROC curve was generated by performing 1000 bootstraps. **B)** Using ROC curve analysis, EC and IC labels were assigned to sequences from conditioned media (EV enrichment) using a specificity threshold of 0.75. These experimental labels were subsequently employed to construct a confusion matrix for the classification of ECX and MUT sequences. Performance metrics are provided for this classification. **C)** The presented contingency table illustrates the experimental distribution of ExoGRU generated REX and RIX sequences in EV. The ExoGRU class predictions for these synthetic sequences achieved an accuracy of 67%, with 59% sensitivity and 77% specificity. A $\chi 2$ test was applied to calculate a p-value for the observed counts (P=0.0005). **D)** Ct Values and Normalized CM Enrichment of REX and RIX Sequences in the CM fraction. All sequences were cloned under a RNA polymerase III promoter, and their expression in CM was initially normalized against mir-16. Subsequently, the values were further normalized against the corresponding expression of the sequences in IC. REX-1 and REX-5 have

significantly lower Ct's than RIX sequences, and they show significantly higher EV enrichment relative to RIX controls, resulting in a combined Fisher's P values of P<1e-100 and P=1e-4 respectively.

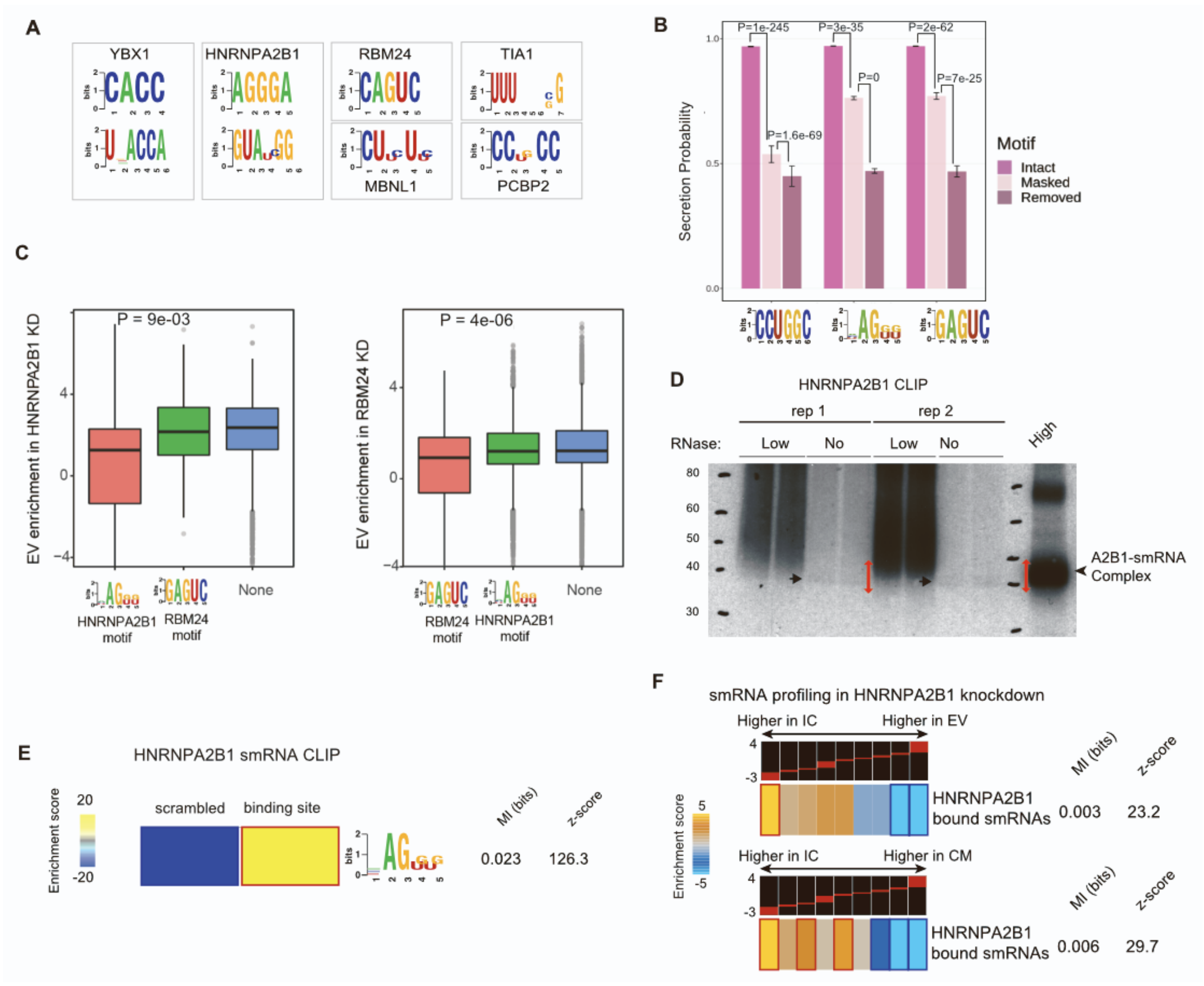

Supplemental Figure 3. ExoGRU RBP motif discovery and CLIPseq to identify small RNA secretion mechanisms, related to figure 3. A) RNA binding protein motifs found by exoGRU to be enriched in ECX sequences and their corresponding RBPs. **B)** Comparative analysis of the model's secretion probability before and after masking (or deleting) enriched RBP motifs in the dataset, including 80 sequences with the CCUGGC motif, 304 sequences with the GAGUC motif, and 1888 sequences with the [ACU]AG[GU][GU] pattern. Each bar in the graph illustrates the average secretion probability before and after the masking or deletion of motifs, with the standard error of the mean (SEM) represented by the error bars atop each bar. Statistical significance was assessed using a Wilcoxon test, comparing the secretion probabilities before and after motif masking. To mask the matched motifs, we set the corresponding inputs to 0 across one-hot encoded channel; to remove the motif, we simply deleted that

portion of the input sequence. **C)** The first graph illustrates the enrichment of small RNAs containing HNRNPA2B1 motifs, RBM24 motifs, or neither in extracellular vesicles isolated from MDA cells with reduced expression of HNRNPA2B1 protein. A notable reduction in the enrichment of small RNAs containing HNRNPA2B1 motifs is observed in comparison to those containing RBM24 motifs (p-value = 9e-03). The second graph depicts a similar analysis but in MDA samples with depleted RBM24 protein. A significant decrease in the enrichment of small RNAs containing RBM24 motifs is observed compared to those containing HNRNPA2B1 motifs (p-value = 4e-06). P-values are calculated using Mann-Whitney U test. The Y axis represents the ratio of EV/IC of these motifs in the KD vs CTRL cell line. **D)** Image of radiolabeled RNA bound to HNRNPA2B1 via CLIP. Protein-RNA complexes were treated with no, low and high RNase after crosslinking. The black arrows point to the faint but visible small RNA band in the no RNAse lane. To retrieve the small RNAs bound by HNRNPA2B1, we excised the membrane in the 40-50kDa range for the no-RNase lanes, which corresponds to the artificially created small RNA-HNRNPA2B1 complex in the high-RNase lane (this range is marked by a double-ended red arrows). **E)** Heatmap illustrates the enrichment of HNRNPA2B1 motifs within HNRNPA2B1 binding sites identified through CLIP-seq, compared to scrambled sequences (with di-nucleotide frequency held constant). Red and bolded borders show statistically significant enrichments, as determined by a hypergeometric test (corrected $P < 0.05$). MI value and associated *z*-score are shown. **F)** Heatmap showing pattern of enrichment or depletion of HNRNPA2B1-bound small RNA sequences (captured by CLIP-seq) in EV and CM fractions upon HNRNPA2B1 knockdown. The panels with black bins show how the sequences are partitioned into equally populated bins based on their EV and CM enrichment measures, going from left (lowly expressed in EV/CM) to right (highly expressed in EV/CM). In the heatmap representation, a gold entry marks the enrichment of the HNRNPA2B1-bound small RNA in its corresponding EV or CM expression bins (measured by log-transformed hypergeometric *P*-values), while a light-blue entry indicates HNRNPA2B1-bound small RNA depletion in the bin. Red and blue borders mark highly significant motif enrichments and depletions, respectively. MI value and associated *z*-score are shown.

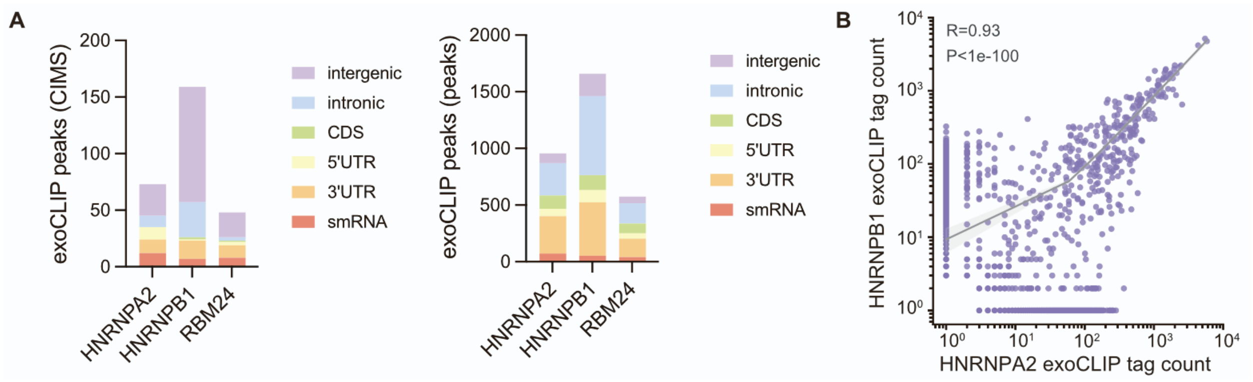

Supplemental Figure 4. Annotated exoCLIP sequencing results, related to figure 4. A) Use of CLIP Toolkit to analyze the HNRNPA2, HNRNPB1 and RBM24 exoCLIP results. For each RNA-binding protein (RBP), two distinct methods were applied to identify peaks. The first plot shows crosslinking induced mutations (CIMs),while the second plot relies on the peak signal or sequence coverage. These plots reveal a substantial abundance of RNA targets associated with each RBP, and these RNA targets were further categorized into various small RNA (smRNA) subtypes.**B)** Scatter plot indicates a high correlation between the distribution of HNRNPA2 and HNRNPB1 tags extracted from exoCLIP data (R=0.93). The plot also shows specific binding sites for these two isoforms.

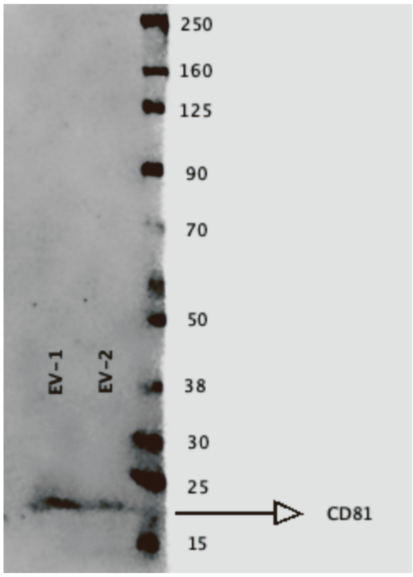

Supplemental Figure 5. Validation of PEG precipitation method in EV isolation, related to the STAR Methods: RNA isolation from conditioned media (CM) and extracellular vesicles (EV). A western blot image illustrating the presence of CD81 protein, a common exosomal marker in EV samples isolated from MDA-MB-231 conditioned media using the PEG precipitation method.
