# Supplementary material for: Revealing the grammar of small RNA secretion using interpretable machine learning
Source: Cell Genom. 2024 Mar 8;4(4):100522. doi: 10.1016/j.xgen.2024.100522 (PMC11019361; doi:10.1016/j.xgen.2024.100522)
Supplement: Document S2. Article plus supplemental information [file mmc11.pdf]

Cell Genomics

Article

Revealing the grammar of small RNA secretion using interpretable machine learning

Graphical abstract

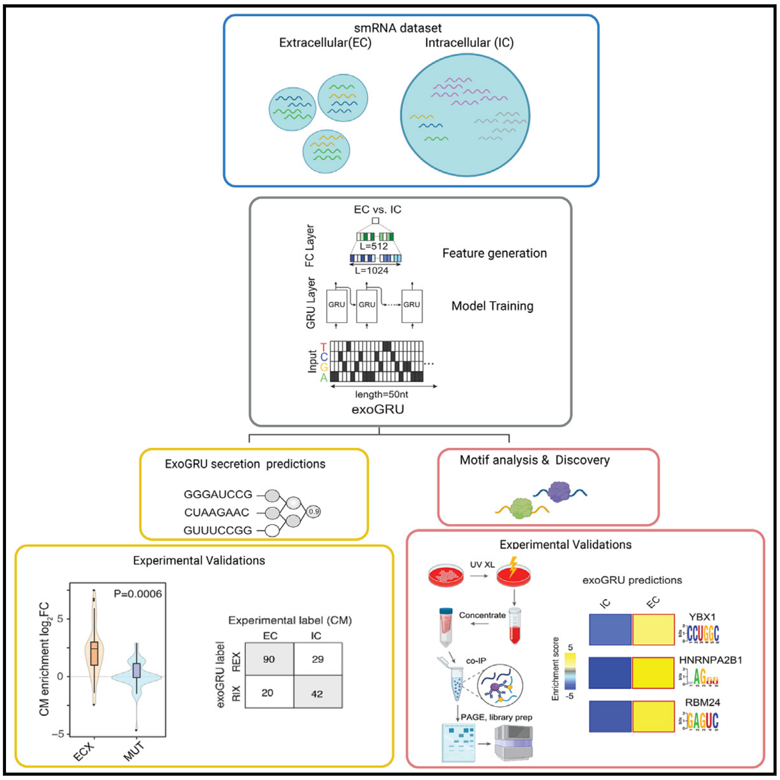

Highlights

- We developed ExoGRU, a machine learning tool for predicting small RNA secretion
- ExoGRU utilizes primary sequences of small RNAs to accurately predict their secretion
- ExoGRU identifies both known and novel RBPs governing small RNA secretion
- Using ExoGRU, we generated synthetic small RNAs with high secretion ability

Authors

Bahar Zirak, Mohsen Naghipourfar, Ali Saberi, ..., Albertas Navickas, Ali Sharifi-Zarchi, Hani Goodarzi

Correspondence

albertas.navickas@curie.fr (A.N.),
asharifiz@gmail.com (A.S.-Z.),
hani.goodarzi@ucsf.edu (H.G.)

In brief

Zirak et al. introduce ExoGRU, a machine learning model predicting small RNA secretion from primary RNA sequences. Additionally, the study introduces exoCLIP, unveiling RNA and RNA-binding protein interactions in the extracellular space. These insights hold promise for therapeutic and synthetic biology applications.

Zirak et al., 2024, Cell Genomics *4*, 100522
April 10, 2024 © 2024 The Authors.
https://doi.org/10.1016/j.xgen.2024.100522

CellPress

Cell Genomics

CellPress
OPEN ACCESS

Article

Revealing the grammar of small RNA secretion using interpretable machine learning

Bahar Zirak,[1,2,3,4,15] Mohsen Naghipourfar,[5,15] Ali Saberi,[6,7,15] Delaram Pouyabahar,[8,9,15] Amirhossein Zarezadeh,[10,11,15] Lixi Luo,[1,2,3,4,12,15] Lisa Fish,[1,2,3,4] Doowon Huh,[13] Albertas Navickas,[1,2,3,4,14,*] Ali Sharifi-Zarchi,[5,*] and Hani Goodarzi[1,2,3,4,16,*]

[1]Department of Biochemistry & Biophysics, University of California, San Francisco, San Francisco, CA, USA
[2]Department of Urology, University of California, San Francisco, San Francisco, CA, USA
[3]Helen Diller Family Comprehensive Cancer Center, University of California, San Francisco, San Francisco, CA, USA
[4]Bakar Computational Health Sciences Institute, University of California, San Francisco, San Francisco, CA, US
[5]Department of Computer Engineering, Sharif University of Technology, Tehran, Iran
[6]Department of Electrical and Computer Engineering, McGill University, Montreal, QC H3A 0E9, Canada
[7]McGill Genome Centre, Victor Phillip Dahdaleh Institute of Genomic Medicine, 740 Dr Penfield Avenue, Montreal, QC H3A 0G1, Canada
[8]Department of Molecular Genetics, University of Toronto, Toronto, ON, Canada
[9]The Donnelly Centre, University of Toronto, Toronto, ON, Canada
[10]Department of Stem Cells and Developmental Biology, Cell Science Research Center, Royan Institute for Stem Cell Biology and Technology, ACECR, Tehran, Iran
[11]Department of Developmental Biology, School of Basic Sciences and Advanced Technologies in Biology, University of Science and Culture, Tehran, Iran
[12]Department of Surgical Oncology, Sir Run Run Shaw Hospital, Zhejiang University School of Medicine, Hangzhou, China
[13]Laboratory of Systems Cancer Biology, The Rockefeller University, New York, NY, USA
[14]Institut Curie, CNRS UMR3348, INSERM U1278, Orsay, France
[15]These authors contributed equally
[16]Lead contact
*Correspondence: albertas.navickas@curie.fr (A.N.), asharifiz@gmail.com (A.S.-Z.), hani.goodarzi@ucsf.edu (H.G.)
https://doi.org/10.1016/j.xgen.2024.100522

SUMMARY

Small non-coding RNAs can be secreted through a variety of mechanisms, including exosomal sorting, in small extracellular vesicles, and within lipoprotein complexes. However, the mechanisms that govern their sorting and secretion are not well understood. Here, we present ExoGRU, a machine learning model that predicts small RNA secretion probabilities from primary RNA sequences. We experimentally validated the performance of this model through ExoGRU-guided mutagenesis and synthetic RNA sequence analysis. Additionally, we used ExoGRU to reveal *cis* and *trans* factors that underlie small RNA secretion, including known and novel RNA-binding proteins (RBPs), e.g., YBX1, HNRNPA2B1, and RBM24. We also developed a novel technique called exoCLIP, which reveals the RNA interactome of RBPs within the cell-free space. Together, our results demonstrate the power of machine learning in revealing novel biological mechanisms. In addition to providing deeper insight into small RNA secretion, this knowledge can be leveraged in therapeutic and synthetic biology applications.

INTRODUCTION

Small non-coding RNAs play a variety of regulatory functions in the cell, including regulation of mRNA stability and protein synthesis.[1,2] However, some small RNAs also reside in the extracellular space, packaged within extracellular vesicles or lipoprotein complexes, for example, where they are thought to play roles in cellular communication.[3–6] Many recent studies have focused on the role of these secreted small RNAs as potential biomarkers in various diseases, particularly cancer.[7–9] RNA secretion, however, is not a random process. While some studies have focused on identifying the various mechanisms through which small RNAs are secreted,[10,11] our knowledge of the underlying regulatory programs that govern extracellular sorting remains incomplete.

To reveal the *cis*-regulatory grammar that underlies small RNA secretion, we developed ExoGRU, a deep-learning model for predicting secretion probabilities of small RNAs based on their primary sequence. In addition to the commonly used machine learning performance metrics, we also used two independent experimental approaches to validate the veracity of our model. We used ExoGRU to (1) identify mutations that abrogate the secretion of known cell-free small RNAs and (2) predict high-confidence sets of synthetic sequences that are secreted or retained. Having confirmed the accuracy of ExoGRU using these experimental strategies, we interrogated the model to reveal the *cis*-regulation RNA

This is an open access article under the CC BY license (http://creativecommons.org/licenses/by/4.0/).

secretion grammar that it has learned. In addition to recapitulating known RNA-binding proteins (RBPs) involved in small RNA sorting, such as YBX1, we also discovered and validated RBM24 as a novel RNA secretory factor. We also developed exoCLIP, a variation of cross-linking immunoprecipitation (CLIP-seq),[12] that reveals RBP-RNA interactions in the cell-free space using UV crosslinking immunoprecipitation followed by high-throughput sequencing. Application of exoCLIP to RBM24 and HNRNPA2B1, another factor that was nominated by our model and previously implicated in RNA secretion, further confirmed their direct interactions with target small RNAs in extracellular vesicles.

Our results collectively show the significance of machine learning in uncovering previously unknown biological mechanisms. In addition to capturing the sequence features that mark small RNAs for secretion, our approach provides readily testable hypotheses around the key *trans* factors involved. This not only deepens our understanding of an intricate biological process but also has practical implications for the design of artificial cell-free RNA species in synthetic biology applications.

RESULTS

ExoGRU, a computational model for accurate prediction of small RNA secretion

To learn the small RNA secretory grammar, we first aggregated, curated, and labeled a large compendium of small RNA datasets in the extracellular (EC) or the intracellular (IC) compartment. These datasets, along with their EC vs. IC labels, were obtained from three distinct sources: (1) a dataset of IC and EC small RNAs (between 18 and 50 nt) we had previously generated across eight cell line models,[7] (2) the Extracellular RNA Communication Consortium Atlas[13] dataset, and (3) The Cancer Genome Atlas small RNA sequencing data.[14] Given that the cell-free RNA content is not correlated with the abundance of small RNAs in the cell, we hypothesized that a *cis*-regulatory grammar serves as a localization signal for small RNA sorting into EC space. First, to search for EC-associated RNA sequence and structural features, we compiled the primary sequence, k-mer frequencies (k = 1, 2, 3, 4, 5, 7), *in silico* folding free energy, and predicted secondary structures as input features to train our model (Figure 1A). Starting with simpler models, we trained linear support vector machines (SVMs), Gaussian kernel SVMs, and random forests as classifiers. The poor performance of these models (maximum area under the receiver operating characteristic [AUC]: 0.71) motivated us to train more complex models with increased learning capacity. We tested various neural network architectures, starting with shallow convolutional neural networks (CNNs) and recurrent neural networks, as well as DeepBind, a previously developed CNN model.[15] Upon hyperparameter tuning, we observed an increase in performance upon switching to a gated recurrent unit (GRU)-based deep recurrent neural network architecture (Figure 1B). As shown in Figure S1A, we benchmarked our GRU model, which we named ExoGRU (Figure 1C), against several existing machine learning and deep-learning models. Figure 1D shows the performance of ExoGRU, evaluated on the held-out test set, in which we achieved an area under ROC of 0.95 and an area under the precision recall curve of 0.8, respectively. At 83% specificity, the sensitivity of ExoGRU was 91% (see the confusion matrix in Figure S1B). We also sought to assess the contribution of each input feature to the performance of ExoGRU. From our initial list of features described above, we observed that the primary sequence alone is sufficient to effectively distinguish IC sequences from EC sequences. Furthermore, we conducted a comparative evaluation between our ExoGRU model and several established RNA localization prediction models. Notably, many of these existing models were primarily designed and trained for long non-coding RNAs, which inherently differ from the shorter small RNAs that we focus on in our study. Nevertheless, we conducted an extensive analysis of our model's quality metrics in comparison to some of the existing models that accept short RNAs as input. As shown in Figure S1C, the results revealed significantly superior performance with ExoGRU.

Experimental verification of ExoGRU predictions

To further evaluate the performance of our model, we sought to focus on small RNAs whose status is predicted by ExoGRU with high confidence, i.e., focusing on high-confidence true positives and negatives. For this, we used ExoGRU to select those sequences with the highest and lowest secretion probabilities and labeled them as ECX (high-confidence EC) and ICX (high-confidence IC) (Figures S1D and S1E). Secretion probabilities are computed from the sigmoid-transformed output of the ExoGRU's predictions. The ECX group consists of accurately predicted EC sequences with a secretion probability exceeding 95%, while the ICX group consists of true IC sequences with a secretion probability below 5%. Therefore, both the ECX and ICX groups, by definition, exclude any falsely predicted sequences. Furthermore, we assessed whether the predictive power of our model was consistent across broad small RNA classes and biotypes. Figure S1F displays similarly strong performance metrics for miRNAs (microRNAs), small nucleolar RNAs, small cytoplasmic RNAs, and tRNAs (transfer RNAs), indicating that ExoGRU is capable of accurately predicting small RNA secretion across all these classes.

We next implemented a variety of approaches to experimentally verify the ability of ExoGRU to capture the small RNA secretory grammar among these sequences. First, we generated an exogenously expressed a small RNA library composed of two different sets of sequences: high-confidence secreted small RNAs (ECX) and mutated variants of ECX (MUT). The latter set of sequences was generated by randomly mutating ECX small RNAs, in one or two positions, so that ExoGRU no longer classified them as secreted RNAs. We cloned this library, containing both ECX and MUT sequences, in a lentiviral construct downstream of a U6 promoter (pLKO.1 backbone).[16] We then transduced the MDA-MB-231 breast cancer cell line, which was among the lines used in our original dataset.[7,13,14] We isolated small RNAs from extracellular vesicle (EV), conditioned medium (CM), and IC fractions of this library and performed small RNA sequencing across all samples in biological replicates. We then aligned the resulting reads to the reference library to assess the abundance of each small RNA in the EC and IC space. It should be noted that expressing small RNAs via an exogenous construct may result in RNA species that (1) are mis-localized and therefore rapidly degraded and (2) lack

CellPress
OPEN ACCESS

A

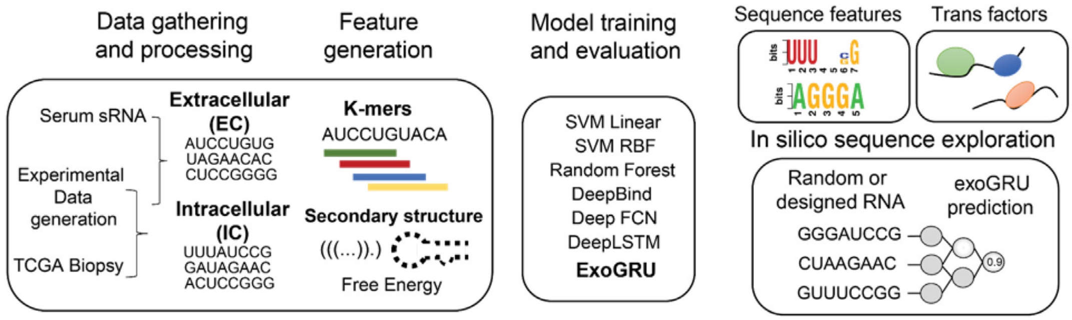

B

Model	Description	Accuracy	Sensitivity	Specificity	Precision	F1
ExoGRU	Best DNN Model: Deep GRU-based Neural Network	0.87	0.91	0.83	0.60	0.72
SVM	Best SVM Model: Primary Sequence - Weighted (1:10) - RBF	0.76	0.55	0.97	0.63	0.59
RF	Best RF Model: Max Tree Depth: 20 - Number of Trees: 50	0.75	0.52	0.97	0.64	0.57

C D

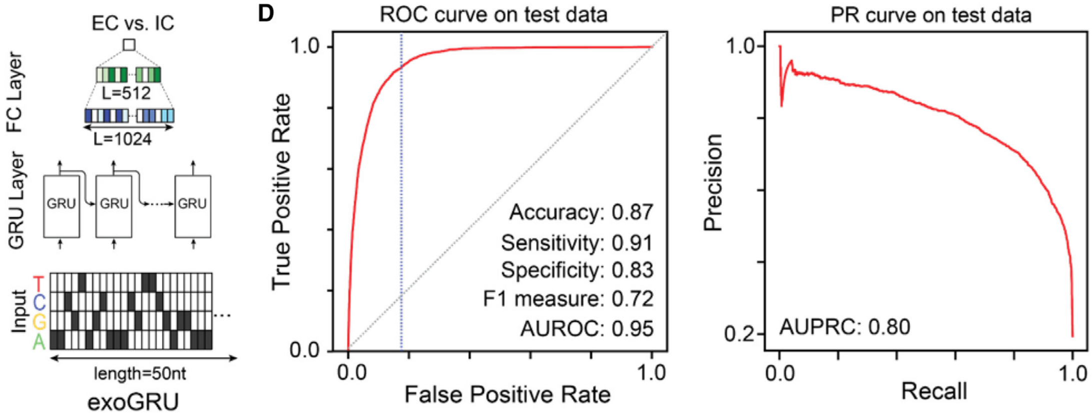

Figure 1. Predicting small RNA (smRNA) secretion from RNA sequence and structural features

(A and B) An overview of our strategy in this study: we used in-house and publicly available data to curate a dataset of intracellular (IC) and cell-free smRNA species. Following extensive feature engineering and evaluating various modeling strategies, we selected the best machine learning models for prediction of smRNA secretion. We observed that ExoGRU, a recurrent neural network model, outperforms other models in this task. We then performed feature attribution scoring and model dissection to dissect the *cis*-regulatory grammar captured by ExoGRU.

(C) The architecture of ExoGRU following hyperparameter optimization.

(D) Receiver operating characteristic (ROC) and precision-recall (PR) curves for the ExoGRU model for the held-out test set. Positive samples are the extracellular (EC) sequences, and negative samples are the IC ones. The performance metrics of this model are also listed.

the endogenous molecular context they rely on for successful secretion. Of the 400 pairs tested, in 55 cases, both the ECX and MUT pairs were stably expressed and therefore successfully captured by our assay. In order to assign a secretion probability to each small RNA, we compared its abundance in the EC fractions (EV or CM) to IC RNA. We observed that the resulting "enrichment scores" were significantly higher for EC small RNAs (ECX), compared to their MUT counterparts, in both CM and EV fractions (Figure 2A). We observed that a large fraction (93%) of exogenously expressed ECX sequences were indeed secreted, and more importantly, slight modifications to these sequences, guided by ExoGRU, resulted in a substantial and significant drop in their secretion potential. To assess the concordance between experimental measurements and ExoGRU predictions, we used a ROC curve to measure the association between experimental and ExoGRU labels at every classification threshold across the CM enrichment score (Figure 2B) and the EV enrichment score (Figure S2A). We used the threshold resulting in a specificity of 0.75 to make EC and IC calls based on the experimental CM enrichment score. We used the resulting experimental classes to generate a confusion matrix against the ExoGRU labels and to calculate performance metrics (Figure 2C). We also performed a similar analysis for the EV fraction, presented in Figure S2B, by calculating an experimental EV enrichment score. Our observations in the EV fraction were similar to the CM fraction, albeit with a lower performance (70% accuracy vs. 82% in CM). This was not unexpected since EV purification often suffers from technical variation and the recovered RNA levels are substantially lower.

The ability of ExoGRU to generalize its predictions to synthetic sequences

We next sought to determine whether ExoGRU can be used for generation, as opposed to mere classification, of synthetic small RNA sequences that are secreted effectively. Furthermore, we sought to assess whether the patterns learned by ExoGRU

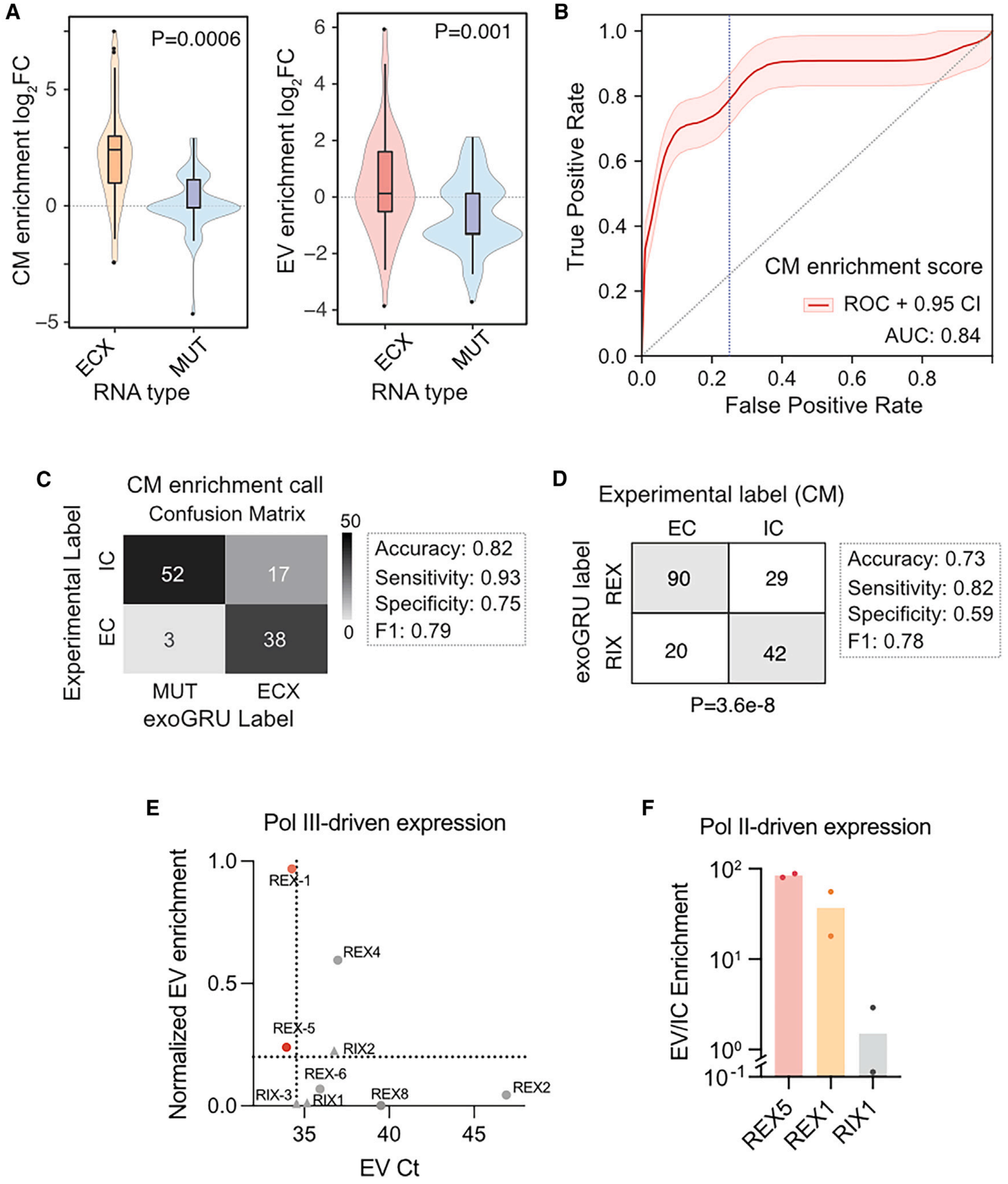

Figure 2. Experimental validations of ExoGRU predictions

(A) Enrichment scores of ECX vs. muted ECX smRNA in conditioned medium (CM) fractions and EV fractions are shown as log2 fold change of smRNA abundances in the EV or CM fraction relative to the IC fraction. A total of 55 ECX and 55 matched mutated (MUT) ECX sequences were successfully expressed and used for this analysis. p values are 0.0006 and 0.001 for CM and EV enrichments, respectively, calculated using Wilcoxon signed-rank test.

(B) ROC curve generated using ECX and MUT experimental CM enrichment scores and ExoGRU's localization predictions to measure the association between the experimental vs. ExoGRU labels at every classification threshold. The smoothened ROC curve was generated by performing 1,000 bootstraps.

(C) EC and IC labels were assigned to sequences from CMs (CM enrichment) using a specificity threshold of 0.75. These experimental labels were subsequently employed to construct a confusion matrix for the classification of ECX and MUT sequences. Performance metrics are provided for this classification.

(D) The presented contingency table illustrates the experimental distribution of ExoGRU-generated REX and RIX sequences in CMs. The ExoGRU class predictions for these synthetic sequences achieved an accuracy of 73%, with 82% sensitivity and 59% specificity. A $\chi 2$ test was applied to calculate a p value for the observed counts ($p = 3.6e-8$).

(E) Ct values and normalized EV enrichment of REX and RIX sequences. All sequences were cloned under an RNA polymerase III promoter, and their expression in EV was initially normalized against mir-16. Subsequently, the values were then corrected by their abundance in the IC fraction. The thresholds on Ct and EV

(legend continued on next page)

based on natural small RNAs are sufficiently generalizable to predict secretion probability of synthetic sequences. To these ends, we randomly generated RNA sequences with an average length of 20 nt and dinucleotide frequencies matching those observed in the endogenous small RNAs. We then used ExoGRU to estimate their probability of secretion and selected ~400 sequences that were classified as EC (labeled REX for randomly generated ECX) and a similar number that were classified as IC (labeled RIX for randomly generated ICX). We synthesized REX/RIX sequences and cloned them similarly to the above. Finally, we transduced this library into MDA-MB-231 cells and profiled small RNAs from the CMs and EVs. If a given randomly generated sequence was observed in the EC fraction, it was given the EC label, otherwise it was labeled as IC. In Figures 2D and S2C, we have provided the resulting contingency table comparing the experimental and computational labels for CM and EV fractions, respectively. The accuracy of ExoGRU class predictions for these synthetic sequences in the CM fraction was 73%, with 82% sensitivity and 59% specificity. We also used a $\chi 2$ test to calculate a p value for the observed counts ($p = 3.6e{-}8$).

We were intrigued by the ability of ExoGRU to generalize well to previously unseen sequences and to effectively identify entirely synthetic sequences that are efficiently secreted. Therefore, to independently verify the patterns observed for the REX and RIX sets in our sequencing data, we selected eight REX sequences (REX1 through REX8) and three RIX sequences (RIX1 through RIX3). We cloned these under an RNA polymerase III (RNA Pol III) promoter (the same pLKO.1 backbone as the library) and generated MDA-MB-231 cell lines for each construct individually. After isolating small RNAs from EV and IC fractions in biological replicates, we performed RT-qPCR to compare the enrichment of each sequence in the EC fraction. We used both the abundance and enrichment of small RNAs in the EV and CM fractions as our selection criteria. We used miR-16, which is abundantly secreted, as an endogenous control in this assay, and both IC and EV or CM values were first normalized to miR-16. REX1 and REX5 small RNAs, which were significantly enriched in the EC fraction based on small RNA sequencing data (corrected p value = 0.033 and p = 0.049, respectively), were further validated as EC-associated small RNAs using targeted RT-qPCR (Figures 2E and S2D). Finally, we also tested the expression and secretion of REX1 and REX5 in MDA-MB-231 cells when cloned under a CMV promoter in the BdLV backbone.[17] To do so, we used self-cleaving ribozymes[18] to express our REX/RIX sequences under this RNA Pol II promoter. In this case as well, we observed a close to a 100-fold enrichment of REX1 and REX5 in the EV fraction (Figure 2F). Together, our results validate the performance and utility of ExoGRU as both a predictive model that captures the small RNA secretory grammar and a generative model that can nominate synthetic small RNAs that are effectively secreted.

Gaining insights into the RNA secretory mechanisms by dissecting the grammar learned by ExoGRU

ExoGRU effectively captures the probability of secretion from the primary RNA sequence alone, which implies the presence of an underlying shared sequence grammar that governs this process. *Cis*-regulatory elements often mediate interactions with master regulators, such as RBP, to influence the RNA life cycle. In fact, several RBPs have already been shown to play a direct role in RNA sorting into exosomes.[3] In order to systematically explore the role of RBPs in small RNA sorting and secretion, we first focused on applying motif discovery methods to the ECX and ICX sequences to find highly discriminative and class-specific motifs. We used three separate motif finding strategies, namely MEME,[19] Homer,[20] and FIRE.[21] We identified multiple sequence motifs that were enriched specifically in the ECX sequences. In parallel, we also used CLIP-seq data from the RNA ENCODE project[22] to identify RBPs whose binding sites are enriched among the secreted RNAs. Using signal and peak-calling results of each RBP, and genome coordinates of ECX and ICX sequences, we sought to identify RBPs that are enriched for interactions with the ECX sequences. We applied the Mann-Whitney statistical test to detect such significantly greater overall signal values among the ECX and ICX regions. In contrast to the motif analysis, ENCODE's eCLIP data resulted in few, if any, leads. This is not surprising since CLIP data originates from longer RNAs that are nuclease treated into shorter cross-linked fragments. As a result, the much stronger signal from longer RNAs largely masks *bona fide* small RNA-RBP interactions. In fact, CLIP analysis for small RNA binding has been reported for only a handful of RBPs, notably AGO2[23] and YBX1.[24] Therefore, for the purpose of this study, we focused our downstream analyses on RBPs with enriched bindings sites (Figure S3A).

Among the RBP motifs enriched in ECX small RNAs, we focused on YBX1, HNRNPA2B1, and RBM24 binding sites since their associated RBPs are also found within the EC space.[25] As shown in Figure 3A, the known motifs for these RBPs were significantly enriched among cell-free small RNAs, even when controlled for length and dinucleotide content. Reidentification of YBX1 through this approach serves as a validation of our strategy given that it is known to be a major factor in miRNA and small RNA sorting into the exosomal compartment.[26] Similarly, while not as well characterized, HNRNPA2B1 has also been previously implicated in miRNA sorting.[27] RBM24, on the other hand, does not have a canonical role in RNA secretion; however, it is known to be present within exosomes.[28] To gain deeper insights into these sequence features used by ExoGRU, we implemented a signal ablation strategy to investigate the influence of masking the identified motifs on the model's predictions. Specifically, we collected approximately 5,000 sequences from the IC and EC datasets that contained matches to our three specified RBP motifs. We subsequently conducted a comparative analysis of the model's mean secretion probabilities before and after masking or completely removing

enrichment axes (shown as dotted lines) are set as one standard deviation from the average of these values for RIX RNAs. REX-1 and REX-5, highlighted in red, satisfy both constraints (based on their *Z* scores relative to RIX sequences), with combined Fisher's p values of 1e−11 and 1e−2, respectively.
(F) Independent validation of EV enrichment for REX1, REX5, and RIX1 sequences expressed under RNA polymerase II promoter. The qPCR analysis was conducted in a manner similar to that depicted in (E).

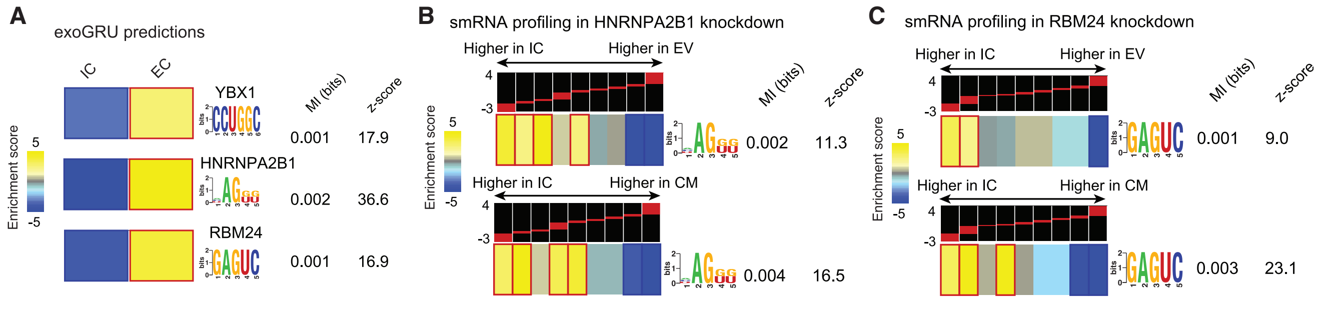

Figure 3. Use of ExoGRU in dissecting RNA secretory mechanisms

(A) As predicted by exoGRU, YBX1, HNRNPA2B1, and RBM24 motifs are enriched in EC. Each RNA structural motif is shown (far right) along with its pattern of enrichment/depletion across the range of RBPs' expression (far left). In the heatmap representation, a gold entry marks the enrichment of the given motif in its corresponding expression bin (measured by log-transformed hypergeometric p values), while a light blue entry indicates motif depletion in the bin. Statistically significant enrichments and depletions are marked with red and dark blue borders, respectively. Also shown are the mutual information (MI) values and their associated *Z* scores.[21] Each MI value is used to calculate a *Z* score, which is the number of standard deviations of the actual MI relative to MIs calculated for randomly shuffled expression profiles. Also shown are the MI values and their associated *Z* scores measuring the association between motif presence and absence and EC enrichment.

(B) Heatmap showing enrichment score of smRNAs containing HNRNPA2B1 motifs in IC, EV, and CM upon decreasing HNRNPA2B1 expression. The log-fold enrichment values were divided into nine equally populated bins, and the enrichment and depletion patterns across the bins were depicted as described in (A). Red and blue borders mark highly significant motif enrichments and depletions, respectively. From left to right, we show the motif names and their sequence information ("motif," in the form of an alphanumeric plot), their associated MI values, and their *Z* scores.

(C) Similar heatmaps showing enrichment score of smRNAs containing RBM24 motifs in IC, EV, and CM upon decreasing RBM24 expression.

these enriched motifs linked to the proteins of interest. Notably, this analysis revealed a substantial reduction in the model's secretion probabilities for all three selected motifs (Figure S3B). Among the previously labeled ECX sequences, more than 94% of them are no longer classified as ECX when the YBX1 motif (CCUGGC) is masked, with an average secretion probability drop from 0.97 to 0.52 (Figure S3B). Additionally, for the RBM24 motif (GAGUC), more than 77% of ECX sequences are no longer predicted as ECX (average secretion probability drop from 0.97 to 0.81). Also, for the HNRNPA2B1 motif ([ACU]AG[GU][GU]), more than 67% of previously ECX-labeled sequences are no longer ECX (average secretion probability drop from 0.97 to 0.76; Figure S3B). These findings were consistent with those from the application of saliency maps and DeepLIFT[29] to sequences containing the specified motifs, reinforcing the crucial role played by these identified motifs in shaping the model's predictions.

To further explore the role of HNRNPA2B1 and RBM24 in small RNA secretion, we used CRISPR interference to knock down these RBPs and measure their consequences on the cell-free RNA content. We achieved a 77% knockdown for HNRNPA2B1 and 88% for RBM24 in MDA-MB-231 cells using lentiviral transduction, as described in the STAR Methods. We then isolated RNA from EV, CM, and IC compartments for small RNA sequencing. As shown in Figures 3B and 3C, silencing HNRNPA2B1 and RBM24 resulted in a significant reduction in the abundance of small RNAs that contained their binding sites in both the EV and CM fractions. This observation confirms the involvement of these RBPs in RNA sorting and secretion. In addition, to further demonstrate the specificity of HNRNPA2B1 and RBM24 for their targets, we grouped the EV enrichment values of small RNAs based on their matches to HNRNPA2B1 and RBM24 motifs, respectively. Figure S3C demonstrates that EV enrichment of small RNAs carrying HNRNPA2B1 and RBM24 motifs was significantly decreased upon knockdown of HNRNPA2B1 and RBM24, respectively. Notably, this decrease was specific to their cognate motifs.

We next sought to confirm that, as previously claimed, HNRNPA2B1 sorts small RNAs it binds into exosomes. For this, we took advantage of UV crosslinking co-immunoprecipitation followed by sequencing. CLIP-seq often includes a nuclease digestion step to footprint RBP binding sites across the transcriptome; however, by omitting this step, the small RNA targets bound by an RBP of interest can be profiled instead. We and others have previously used this approach for other RBPs, such as AGO2[23] and YBX1.[24] Visualization of radiolabeled RNA crosslinked to HNRNPA2B1 on a denaturing gel revealed a faint but visible band at the correct size range (Figure S3D). We extracted these HNRNPA2B1-bound RNAs and performed high-throughput sequencing. Motif analysis of the identified binding site showed a strong and highly significant enrichment of the HNRNPA2B1 motif among the bound small RNAs (Figure S3E), which serves as a technical quality control. Finally, we asked whether these HNRNPA2B1-bound small RNAs were among those depleted from the exosomal space upon HNRNPA2B1 knockdown. Consistently, we observed a marked reduction in the secretion of these RNA, with a higher statistical significance compared to the HNRNPA2B1 motif analysis (Figure S3F). Together with the prior reports, our results show that HNRNPA2B1 binding to small RNAs is required for their effective secretion.

HNRNPA2B1 and RBM24 exoCLIP shows enrichment of EC predicted sequences

The presence of RBPs HNRNPA2B1 and RBM24 in EVs along with their putative small RNA targets strongly suggests direct interactions within the exosomal space. However, direct evidence of RNA binding and the identity of their target RNAs remained lacking. To tackle this problem, we developed a novel approach for capturing the specific RNA molecules that a given RBP interacts with in the exosomal space. This approach, which we have named exoCLIP, is similar to CLIP-seq but uses UV treatment of CMs to crosslink RBP-RNA complexes in the cell-free fraction (Figure 4A).

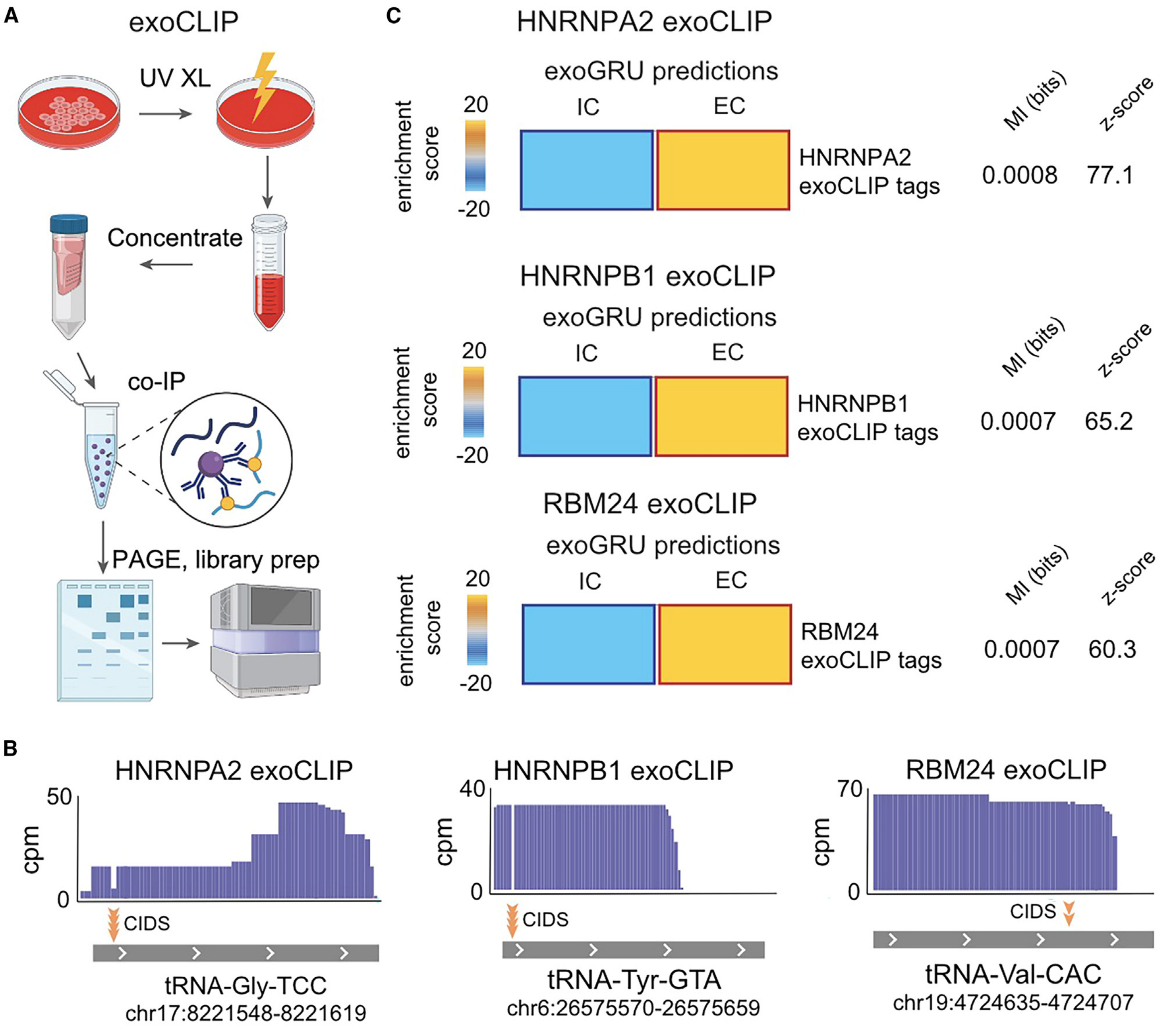

Figure 4. Applying exoCLIP to look at the enrichment of HNRNPA2B1- and RBM24-bound smRNA sequences in cell-free media

(A) Overview of exoCLIP workflow: UV treatment of CMs to crosslink RBP-RNA complexes and using co-immunoprecipitation (coIP) to pull down the RBP-RNA complexes of interest followed by RNA library preparation and sequencing.

(B) Examples of tRNA fragments that are associated with HNRNPA2, HNRNPB1, and RBM24 proteins, as extracted from exoCLIP data. The positions of crosslinking-induced deletions (CIDs) are also highlighted in each case by the yellow arrows. In total, the HNRNPA2 exoCLIP yielded 34 unique reads, with 23 of them exhibiting CIDs at a statistically significant level ($p = 0$). The HNRNPB1 and RBM24 exoCLIPs each resulted in 88 unique reads, where 87 reads from HNRNPB1 and 2 reads from RBM24 showed CIDs ($p = 0$). p values are calculated by the CTK package.[30]

(C) Heatmaps illustrate enrichment levels of ExoGRU-predicted EC and IC smRNAs in smRNA targets extracted from HNRNPA2, HNRNPB1, and RBM24 exoCLIPs. Red and bolded borders show statistically significant enrichments, as determined by a hypergeometric test (corrected $p < 0.05$). MI value and associated Z score are shown.

Using exoCLIP, we sought to demonstrate a direct interaction between HNRNPA2B1 and RBM24 and their target small RNAs. In the case of HNRNPA2B1, we tested both the A2 and B1 isoforms. We transduced MDA-MB-231 cells with FLAG-tagged copies of HNRNPA2, HNRNPB1, and RBM24, respectively. We then performed exoCLIP-seq for each line using FLAG co-immunoprecipitation. We used the CLIP Toolkit[30] to call peaks for each of the RBPs using two strategies: one based on sequence coverage or signal and the other based on crosslinking-induced mutations. Both strategies yielded between hundreds and thousands of RNA targets, a fraction of which mapped to annotated small RNAs (Figure S4A). These results indicate that HNRNPA2B1 and RBM24 indeed bind their RNA targets directly in the cell-free space. Interestingly, while we observed some correlation between the HNRNPA2 and HNRNPB1 isoforms, there were also many isoform-specific binding sites for these RBPs (Figure S4B). In

Table 1. Breakdown of number of samples used from each dataset

Label	Split	Number of data
EV	train	26,374
EV	valid	3,271
EV	test	3,437
IC	train	114,340
IC	valid	14,267
IC	test	14,270

Figure 4B, we have included examples of small RNAs, in this case tRNA fragments, that are bound by each RBP, as evidenced by the exoCLIP signal and the presence of crosslinking-induced deletions. Since we had selected HNRNPA2B1 and RBM24 based on our analysis of high-confidence predictions for EC and IC RNAs from ExoGRU, we expected these predictions to match the exoCLIP results as well. To assess this possibility, we measured the enrichment of bound small RNAs from each dataset among the ExoGRU-predicted EC vs. IC small RNAs. As shown in Figure 4C, we observed a significant over-representation of EC small RNAs that are directly bound by HNRNPA2B1 and RBM24.

DISCUSSION

It is hypothesized that EC small RNAs play a key role in intercellular communications and regulation of various biological processes.[3,5,6,31,32] Identifying these specific RNA molecules and understanding their mechanisms of action has led to the discovery of different disease-associated biomarkers and therapeutic targets.[7–9,31,33–35] However, our understanding of how these RNA molecules are sorted and delivered into the EC space is still limited.

Multiple studies have identified different RBPs responsible for RNA secretion into the EC space.[26,27] However, the full mechanisms underlying small RNA delivery are still largely unknown. A recent study comparing IC vs. EC miRNA profiles found multiple "EXOmotifs" and "CELLmotifs" on miRNA responsible for their secretion from or retention in the cells, suggesting that there are various different motifs and RBPs involved in this process.[10] While the study provided valuable information on miRNA distribution in metabolically important cells, we aimed to further explore the mechanisms behind small RNA sorting in cancer cells using machine learning tools and novel molecular biology approaches.

To further decipher the principles of small RNA delivery to the EC space, we asked three specific questions: (1) which RNA sequences are selected and secreted, (2) can we develop a computational model that learns the sequence grammar that underlies RNA secretion, and (3) using this model, can we learn the molecular mechanisms that drive this selection? To tackle these questions, we developed ExoGRU, a deep recurrent neural network, to predict the secretion probability for any small RNA given the primary sequence. We rigorously verified our model's ability to capture the small RNA secretory grammar by testing the impact of ExoGRU-guided targeted mutations on the secretion of endogenous small RNAs. We found the RNA primary sequence to be sufficient to discriminate between the IC and EC small RNAs.

Table 2. Number of smRNA sequences categorized as EC(X) and IC(X) and their corresponding secretion probability as predicted by ExoGRU

True label	Secretion probability	Prediction label	Label type	Number of sequences
EC	<0.5	IC	false negative	2,970
EC	≥0.5	EC	true positive	30,112
EC	≥0.95	EC (ECX)	true positive (extreme)	8,944
IC	≥0.5	EC	false positive	20,192
IC	<0.5	IC	true negative	122,685
IC	≤0.05	IC (ICX)	true negative (extreme)	82,157

Additionally, we used ExoGRU to reveal the regulatory grammar captured by the model. Using motif discovery methods and CLIP-seq data combined with high-confidence ExoGRU predictions, we identified several RBPs that preferentially bind to secreted RNAs and are associated with the RNA-sorting process. In addition to recapitulating the known involvement of YBX1, we also demonstrated the role of RBM24 and HNRNPA2B1 in RNA secretion through CRISPR interference and CLIP-seq. We described exoCLIP, a novel approach to capture direct RBP-RNA interactions in cell-free media. Using this method, we successfully characterized RBM24, HNRNPA2, and HNRNPB1 RNA targets in the EC space. Our exoCLIP-seq data also aligned with ExoGRU predictions, as we saw enrichment of EC-associated sequences in these data. Overall, our results demonstrate the performance and utility of ExoGRU as a predictive model that captures the small RNA secretory grammar and provides insights into the role of RBPs in small RNA sorting and secretion.

Last but not least, we showed that ExoGRU's prediction ability is generalizable to synthetic sequences. This was demonstrated through sequencing and qPCR analysis of randomly generated but ExoGRU-scored libraries of EC and IC sequences (REX/RIX). The validation process further confirmed the accuracy of the predictions made by the ExoGRU model. Using this feature of ExoGRU, we will be able to design fully engineered and efficiently secreted sequences that can be used as biomarkers as well as having further applications in synthetic biology.

Limitations of the study

One of the challenges in the biological validation of ExoGRU's findings stems from the expression of small RNA through an exogenous construct. This approach may yield RNA species that (1) experience mis-localization and subsequent rapid degradation and (2) lack the crucial endogenous molecular context required for successful secretion. Consequently, not all sequences confidently identified by ExoGRU will be expressed correctly and captured in the EC domain. Moreover, the experimental isolation process and sequencing threshold may not efficiently capture lowly abundant secreted RNA, leading to mis-labeling these RNA species as IC.

The small RNA composition is significantly variable across diverse cell types. Our model was trained on small RNA derived from breast cancer or normal breast tissue, with validation

CellPress
OPEN ACCESS

exclusively performed on the MDA-MB-231 cell line. While certain discovered sequences may find expression and validation in other cell types, it is crucial to acknowledge the potential limitations. The model's ability to reproduce similar results may be compromised, emphasizing the need for retraining on new datasets that align more closely with the specific context.

STAR★METHODS

Detailed methods are provided in the online version of this paper and include the following:

- KEY RESOURCES TABLE
- RESOURCE AVAILABILITY
  - Lead contact
  - Materials availability
  - Data and code availability
- METHOD DETAILS
  - Cell culture
  - MDA-MB-231 cells with RBP knockdowns
  - MDA-MB-231 cells overexpressing flag-tagged RBPs
  - MDA-MB-231 cells expressing ECX or MUT sequences under pol III promoter
  - MDA-MB-231 cells expressing REX or RIX sequences under pol III promoter
  - MDA-MB-231 cells expressing REX or RIX sequences under pol II promoter
  - RT-qPCR for REX/RIX expression
  - Generation of smRNA libraries
  - RNA isolation from conditioned media (CM) and extracellular vesicles (EV)
  - RNA isolation from cells
  - ExoCLIP
  - Low salt wash buffer
  - Western Blotting
  - Protein K digest and RNA capture
  - Small RNA library preparation
  - Sequencing and analysis
  - HITS-CLIP
  - Data Acquisition
  - Data preprocessing
  - Feature generation
  - Predictive models
  - Support Vector Machines
  - Random Forest
  - ExoGRU
  - ExoLSTM
  - ExoCNN
  - Model training
  - Motif discovery and enrichment
  - Motif comparison
  - RBP binding sites analysis
  - Model Interpretability

SUPPLEMENTAL INFORMATION

Supplemental information can be found online at https://doi.org/10.1016/j.xgen.2024.100522.

ACKNOWLEDGMENTS

We thank Dr. Sohail Tavazoie for his support and his comments on this manuscript. We also thank Drs. Babak Alipanahi and Hamed Najafabadi for their feedback on the earlier versions of this study. We would like to acknowledge the UCSF Center for Advanced Technology (CAT) for high-throughput sequencing and other genomic analyses. Lastly, we would like to acknowledge that the graphical abstract was created with BioRender.com.

AUTHOR CONTRIBUTIONS

Conceptualization, H.G., A.S.-Z., and A.N.; data curation; M.N., A.S., D.P., and A.Z.; formal analysis, M.N., A.S., D.P., A.Z., H.G., B.Z., and L.L.; funding acquisition, H.G.; investigation, B.Z., L.L., M.N., A.S., D.P., A.Z., and D.H.; methodology, M.N., A.S., D.P., A.Z., L.F., H.G., A.S.-Z., and A.N.; project administration, H.G., A.S.-Z., and A.N.; resources, H.G.; software, M.N., A.S., D.P., A.Z., and H.G.; supervision, H.G., A.S.-Z., and A.N.; validation, B.Z., L.L., M.N., A.S., D.P., A.Z., and D.H.; visualization, M.N., A.S., D.P., A.Z., H.G., and B.Z.; writing – original draft, B.Z., M.N., A.S., D.P., H.G., and A.S.-Z.; writing – review & editing, B.Z., M.N., A.S., and H.G.

DECLARATION OF INTERESTS

The authors declare no competing interests.

Received: April 18, 2023
Revised: November 2, 2023
Accepted: February 12, 2024
Published: March 8, 2024

REFERENCES

1. Shimoni, Y., Friedlander, G., Hetzroni, G., Niv, G., Altuvia, S., Biham, O., and Margalit, H. (2007). Regulation of gene expression by small non-coding RNAs: a quantitative view. Mol. Syst. Biol. *3*, 138. https://doi.org/10.1038/msb4100181.
2. Patil, V.S., Zhou, R., and Rana, T.M. (2014). Gene regulation by non-coding RNAs. Crit. Rev. Biochem. Mol. Biol. *49*, 16–32. https://doi.org/10.3109/10409238.2013.844092.
3. O'Brien, K., Breyne, K., Ughetto, S., Laurent, L.C., and Breakefield, X.O. (2020). RNA delivery by extracellular vesicles in mammalian cells and its applications. Nat. Rev. Mol. Cell Biol. *21*, 585–606. https://doi.org/10.1038/s41580-020-0251-y.
4. Sork, H., Conceicao, M., Corso, G., Nordin, J., Lee, Y.X.F., Krjutskov, K., Orzechowski Westholm, J., Vader, P., Pauwels, M., Vandenbroucke, R.E., et al. (2021). Profiling of Extracellular Small RNAs Highlights a Strong Bias towards Non-Vesicular Secretion. Cells *10*, 1543. https://doi.org/10.3390/cells10061543.
5. Mittelbrunn, M., and Sánchez-Madrid, F. (2012). Intercellular communication: diverse structures for exchange of genetic information. Nat. Rev. Mol. Cell Biol. *13*, 328–335. https://doi.org/10.1038/nrm3335.
6. Chen, X., Liang, H., Zhang, J., Zen, K., and Zhang, C.-Y. (2012). Secreted microRNAs: a new form of intercellular communication. Trends Cell Biol. *22*, 125–132. https://doi.org/10.1016/j.tcb.2011.12.001.
7. Fish, L., Zhang, S., Yu, J.X., Culbertson, B., Zhou, A.Y., Goga, A., and Goodarzi, H. (2018). Cancer cells exploit an orphan RNA to drive metastatic progression. Nat. Med. *24*, 1743–1751. https://doi.org/10.1038/s41591-018-0230-4.
8. Badowski, C., He, B., and Garmire, L.X. (2022). Blood-derived lncRNAs as biomarkers for cancer diagnosis: the Good, the Bad and the Beauty. npj Precis. Oncol. *6*, 40. https://doi.org/10.1038/s41698-022-00283-7.
9. Pardini, B., Sabo, A.A., Birolo, G., and Calin, G.A. (2019). Noncoding rnas in extracellular fluids as cancer biomarkers: the new frontier of liquid biopsies. Cancers *11*, 1170. https://doi.org/10.3390/cancers11081170.

10. Garcia-Martin, R., Wang, G., Brandão, B.B., Zanotto, T.M., Shah, S., Kumar Patel, S., Schilling, B., and Kahn, C.R. (2022). MicroRNA sequence codes for small extracellular vesicle release and cellular retention. Nature *601*, 446–451. https://doi.org/10.1038/s41586-021-04234-3.

11. Tosar, J.P., Gámbaro, F., Sanguinetti, J., Bonilla, B., Witwer, K.W., and Cayota, A. (2015). Assessment of small RNA sorting into different extracellular fractions revealed by high-throughput sequencing of breast cell lines. Nucleic Acids Res. *43*, 5601–5616. https://doi.org/10.1093/nar/gkv432.

12. Ule, J., Jensen, K.B., Ruggiu, M., Mele, A., Ule, A., and Darnell, R.B. (2003). CLIP identifies Nova-regulated RNA networks in the brain. Science *302*, 1212–1215. https://doi.org/10.1126/science.1090095.

13. Ainsztein, A.M., Brooks, P.J., Dugan, V.G., Ganguly, A., Guo, M., Howcroft, T.K., Kelley, C.A., Kuo, L.S., Labosky, P.A., Lenzi, R., et al. (2015). The NIH extracellular RNA communication consortium. J. Extracell. Vesicles *4*, 27493. https://doi.org/10.3402/jev.v4.27493.

14. Grossman, R.L., Heath, A.P., Ferretti, V., Varmus, H.E., Lowy, D.R., Kibbe, W.A., and Staudt, L.M. (2016). Toward a shared vision for cancer genomic data. N. Engl. J. Med. *375*, 1109–1112. https://doi.org/10.1056/NEJMp1607591.

15. Alipanahi, B., Delong, A., Weirauch, M.T., and Frey, B.J. (2015). Predicting the sequence specificities of DNA- and RNA-binding proteins by deep learning. Nat. Biotechnol. *33*, 831–838. https://doi.org/10.1038/nbt.3300.

16. Stewart, S.A., Dykxhoorn, D.M., Palliser, D., Mizuno, H., Yu, E.Y., An, D.S., Sabatini, D.M., Chen, I.S.Y., Hahn, W.C., Sharp, P.A., et al. (2003). Lentivirus-delivered stable gene silencing by RNAi in primary cells. RNA *9*, 493–501. https://doi.org/10.1261/rna.2192803.

17. Amendola, M., Venneri, M.A., Biffi, A., Vigna, E., and Naldini, L. (2005). Coordinate dual-gene transgenesis by lentiviral vectors carrying synthetic bidirectional promoters. Nat. Biotechnol. *23*, 108–116. https://doi.org/10.1038/nbt1049.

18. Gao, Y., and Zhao, Y. (2014). Self-processing of ribozyme-flanked RNAs into guide RNAs in vitro and in vivo for CRISPR-mediated genome editing. J. Integr. Plant Biol. *56*, 343–349. https://doi.org/10.1111/jipb.12152.

19. Bailey, T.L., and Elkan, C. (1994). Fitting a mixture model by expectation maximization to discover motifs in biopolymers. Proc. Int. Conf. Intell. Syst. Mol. Biol. *2*, 28–36.

20. Heinz, S., Benner, C., Spann, N., Bertolino, E., Lin, Y.C., Laslo, P., Cheng, J.X., Murre, C., Singh, H., and Glass, C.K. (2010). Simple combinations of lineage-determining transcription factors prime cis-regulatory elements required for macrophage and B cell identities. Mol. Cell *38*, 576–589. https://doi.org/10.1016/j.molcel.2010.05.004.

21. Elemento, O., Slonim, N., and Tavazoie, S. (2007). A universal framework for regulatory element discovery across all genomes and data types. Mol. Cell *28*, 337–350. https://doi.org/10.1016/j.molcel.2007.09.027.

22. Van Nostrand, E.L., Freese, P., Pratt, G.A., Wang, X., Wei, X., Xiao, R., Blue, S.M., Chen, J.-Y., Cody, N.A.L., Dominguez, D., et al. (2020). A large-scale binding and functional map of human RNA-binding proteins. Nature *583*, 711–719. https://doi.org/10.1038/s41586-020-2077-3.

23. Chi, S.W., Zang, J.B., Mele, A., and Darnell, R.B. (2009). Argonaute HITS-CLIP decodes microRNA-mRNA interaction maps. Nature *460*, 479–486. https://doi.org/10.1038/nature08170.

24. Goodarzi, H., Liu, X., Nguyen, H.C.B., Zhang, S., Fish, L., and Tavazoie, S.F. (2015). Endogenous tRNA-Derived Fragments Suppress Breast Cancer Progression via YBX1 Displacement. Cell *161*, 790–802. https://doi.org/10.1016/j.cell.2015.02.053.

25. Keerthikumar, S., Chisanga, D., Ariyaratne, D., Al Saffar, H., Anand, S., Zhao, K., Samuel, M., Pathan, M., Jois, M., Chilamkurti, N., et al. (2016). ExoCarta: A Web-Based Compendium of Exosomal Cargo. J. Mol. Biol. *428*, 688–692. https://doi.org/10.1016/j.jmb.2015.09.019.

26. Shurtleff, M.J., Temoche-Diaz, M.M., Karfilis, K.V., Ri, S., and Schekman, R. (2016). Y-box protein 1 is required to sort microRNAs into exosomes in cells and in a cell-free reaction. Elife *5*, e19276. https://doi.org/10.7554/eLife.19276.

27. Villarroya-Beltri, C., Gutiérrez-Vázquez, C., Sánchez-Cabo, F., Pérez-Hernández, D., Vázquez, J., Martin-Cofreces, N., Martinez-Herrera, D.J., Pascual-Montano, A., Mittelbrunn, M., and Sánchez-Madrid, F. (2013). Sumoylated hnRNPA2B1 controls the sorting of miRNAs into exosomes through binding to specific motifs. Nat. Commun. *4*, 2980. https://doi.org/10.1038/ncomms3980.

28. He, M., Qin, H., Poon, T.C.W., Sze, S.-C., Ding, X., Co, N.N., Ngai, S.-M., Chan, T.-F., and Wong, N. (2015). Hepatocellular carcinoma-derived exosomes promote motility of immortalized hepatocyte through transfer of oncogenic proteins and RNAs. Carcinogenesis *36*, 1008–1018. https://doi.org/10.1093/carcin/bgv081.

29. Shrikumar, A., Greenside, P., and Kundaje, A. (2017). Learning Important Features Through Propagating Activation Differences. Preprint at arXiv. https://doi.org/10.48550/arxiv.1704.02685.

30. Shah, A., Qian, Y., Weyn-Vanhentenryck, S.M., and Zhang, C. (2017). CLIP Tool Kit (CTK): a flexible and robust pipeline to analyze CLIP sequencing data. Bioinformatics *33*, 566–567. https://doi.org/10.1093/bioinformatics/btw653.

31. Sadik, N., Cruz, L., Gurtner, A., Rodosthenous, R.S., Dusoswa, S.A., Ziegler, O., Van Solinge, T.S., Wei, Z., Salvador-Garicano, A.M., Gyorgy, B., et al. (2018). Extracellular rnas: A new awareness of old perspectives. Methods Mol. Biol. *1740*, 1–15. https://doi.org/10.1007/978-1-4939-7652-2_1.

32. Sohail, A.M., Khawar, M.B., Afzal, A., Hassan, A., Shahzaman, S., and Ali, A. (2022). Multifaceted roles of extracellular RNAs in different diseases. Mil. Med. Res. *9*, 43. https://doi.org/10.1186/s40779-022-00405-z.

33. Pita, T., Feliciano, J.R., and Leitão, J.H. (2020). Extracellular RNAs in Bacterial Infections: From Emerging Key Players on Host-Pathogen Interactions to Exploitable Biomarkers and Therapeutic Targets. Int. J. Mol. Sci. *21*, 9634. https://doi.org/10.3390/ijms21249634.

34. Wu, D., Tao, T., Eshraghian, E.A., Lin, P., Li, Z., and Zhu, X. (2022). Extracellular RNA as a kind of communication molecule and emerging cancer biomarker. Front. Oncol. *12*, 960072. https://doi.org/10.3389/fonc.2022.960072.

35. Ilieva, M., and Uchida, S. (2022). Extracellular RNAs as communicators in cardiovascular disease: a narrative review. ExRNA *4*, 14. https://doi.org/10.21037/exrna-22-3.

36. Horlbeck, M.A., Gilbert, L.A., Villalta, J.E., Adamson, B., Pak, R.A., Chen, Y., Fields, A.P., Park, C.Y., Corn, J.E., Kampmann, M., and Weissman, J.S. (2016). Compact and highly active next-generation libraries for CRISPR-mediated gene repression and activation. Elife *5*, e19760. https://doi.org/10.7554/eLife.19760.

37. EP2839278B1 - Methods for exosome isolation - Google Patents. https://patents.google.com/patent/EP2839278B1/en.

38. Smith, T., Heger, A., and Sudbery, I. (2017). UMI-tools: modeling sequencing errors in Unique Molecular Identifiers to improve quantification accuracy. Genome Res. *27*, 491–499. https://doi.org/10.1101/gr.209601.116.

39. Martin, M. (2011). Cutadapt removes adapter sequences from high-throughput sequencing reads. EMBnet. J. *17*, 10. https://doi.org/10.14806/ej.17.1.200.

40. Li, H., and Durbin, R. (2009). Fast and accurate short read alignment with Burrows-Wheeler transform. Bioinformatics *25*, 1754–1760. https://doi.org/10.1093/bioinformatics/btp324.

41. Licatalosi, D.D., Mele, A., Fak, J.J., Ule, J., Kayikci, M., Chi, S.W., Clark, T.A., Schweitzer, A.C., Blume, J.E., Wang, X., et al. (2008). HITS-CLIP yields genome-wide insights into brain alternative RNA processing. Nature *456*, 464–469. https://doi.org/10.1038/nature07488.

42. Simonyan, K., and Zisserman, A. (2014). Very Deep Convolutional Networks for Large-Scale Image Recognition. Preprint at arXiv. https://doi.org/10.48550/arxiv.1409.1556.

43. Niculescu-Mizil, A., and Caruana, R. (2005). Predicting good probabilities with supervised learning. In Proceedings of the 22nd international conference on Machine learning - ICML '05 (ACM Press), pp. 625–632. https://doi.org/10.1145/1102351.1102430.

CellPress
OPEN ACCESS

STAR★METHODS

KEY RESOURCES TABLE

REAGENT or RESOURCE	SOURCE	IDENTIFIER
**Antibodies**		
anti-HNRNPA2B1 antibody for HITS CLIP	Thermo Fisher	Cat# PA5-34939; RPID: AB_2552288
CD81 (B-11)	Santa Cruz Biotechnology	Cat# sc-166029; RPID: AB_2275892
IRDye® 800CW Goat anti-Mouse IgG Secondary Antibody	Licor	Cat# 926–32210; RPID: AB_621842
**Bacterial and virus strains**		
MegaX electrocompetent cells	Thermo Fisher	C640003
NEB Stable Competent E.coli	New England Biolabs	C3040H
**Chemicals, peptides, and recombinant proteins**		
exosome depleted FBS	Thermo Fisher	A2720801
TransIT-Lenti Transfection Reagent	Mirus bio	6604
Polyethylene Glycol 10000 (PEG)	Hampton Research	HR2-607
SMARTer smRNA-Seq Kit for Illumina	Takara	635029
Centricon® Plus Centrifugal Filter	Millipore sigma	UFC701008
Quick-cfRNA Serum & Plasma Kit	Zymo Research	R1059
Quick-RNA Microprep Kit (cat#)	Zymo Research	R1051
anti-flag magnetic beads	Thermo Fisher	A36797
IGEPAL CA-630	Sigma Aldrich	I8896-50ML
**Deposited data**		
Random smRNA library	GEO	NCBI GEO: GSE230012
Endogenous smRNA library	GEO	NCBI GEO: GSE230012
ExoCLIP	GEO	NCBI GEO: GSE230012
RBP_KD smRNA libraries	GEO	NCBI GEO: GSE230012
**Experimental models: Cell lines**		
MDA-MB-231	ATCC	HTB-26
HEK293T	ATCC	CRL-3216
**Oligonucleotides**		
Oligo lists	This paper, Tables S1, S3, S5, S6, and S7	N/A
gBlocks	This paper, Tables S2 and S4	N/A
Indices	This paper, Tables S8 and S9	N/A
**Recombinant DNA**		
pHR-UCOE-EF1a-dCas9-HAxNLS-XTEN80-KRAB-p2a-mCherry	This paper, Backbone: PHR; Addgene	N/A
pLX302-EF1a-RBM24-flag	This paper, backbone pLX302, Addgene	N/A
pLX302-EF1a-HNRNPA2-flag	This paper, backbone pLX302, Addgene	N/A
pLX302-EF1a-HNRNPB1-flag	This paper, backbone pLX302, Addgene	N/A
pLKO.1 puro	Addgene	8453
BdLV_Puro_mCherry	This paper	N/A
BdLV_Puro_mCherry_RGR_REX/RIX	This paper	N/A
**Software and algorithms**		
All codes developed	https://doi.org/10.5281/zenodo.10553402	N/A
MEME	Bailey et al.[19]	N/A
Homer	Heinz et al.[20]	N/A
Fire	Elemento et al.[21]	N/A
CTK	Shah et al.[30]	N/A

RESOURCE AVAILABILITY

Lead contact

Requests for further information and resources should be directed to and will be fulfilled by the lead contact, Hani Goodarzi (hani.goodarzi@ucsf.edu).

Materials availability

This study did not generate any new unique reagents or materials to report.

Data and code availability

The small RNA sequencing data and exoCLIP are available in the Gene Expression Omnibus database (NCBI GEO: GSE230012). The code used in this study is available at https://doi.org/10.5281/zenodo.10553402. The code was developed using the Python and R programming language. The code was designed to reproduce the analyses presented in this manuscript and may be useful for researchers wishing to extend or replicate our findings. The repository includes documentation and instructions on how to use the code.

METHOD DETAILS

Cell culture

All cells were cultured in a 37°C 5% CO2 humidified incubator. The MDA-MB-231 (ATCC HTB-26) breast cancer cell line, and 293T cells (ATCC CRL-3216) were cultured in DMEM high-glucose medium supplemented with 10% FBS, penicillin, streptomycin, and amphotericin B.

All the lentiviral constructs were co-transfected with pCMV-dR8.91 and pMD2.G plasmids using TransIT-Lenti (Mirus) into 293T cells, following manufacturer's protocol. Virus was harvested 48 h post-transfection and passed through a 0.45 μm filter, and added to target cells 24 h after they were seeded.

MDA-MB-231 cells with RBP knockdowns

Gene knockdowns were performed by first transducing MDA-MB-231 with dCas9-KRAB construct via lentiviral delivery of: pHR-U-COE-EF1a-dCas9-HAxNLS-XTEN80-KRAB-p2a-mCherry. MDA-dCas9-KRAB expressing cells were then sorted by FACS isolation of mCherry-positive cells. Guide RNA sequences for CRISPRi-mediated gene knockdown were cloned into pCRISPRia-v2 (Addgene #84832)[36] via BstXI-BlpI sites (see Table S1 for sgRNA sequences). After transduction with sgRNA lentivirus, MDA-MB-231 cells were selected with 2 μg/mL puromycin (Gibco). Knockdown of target genes was assessed by reverse transcription of total RNA to cDNA (Maxima H Minus RT, Thermo), then using sequence specific primers along with PerfeCTa SYBR Green SuperMix (QuantaBio) per the manufacturer's instruction. HPRT was used as an endogenous control (see Table S1 for primer sequences).

MDA-MB-231 cells overexpressing flag-tagged RBPs

For generation of flag tagged RBP cell lines, we cloned gblocks containing RBM24, HNRNPA2 or HNRNPB1 and the flag sequences into pLX302-EF1a plasmid via PacI-NheI sites (Table S2 shows the gblock sequences). Plasmids were delivered to MDA-MB-231 by lentiviral transduction as described above. Expression of RBP-FLAG was assessed using western blot.

MDA-MB-231 cells expressing ECX or MUT sequences under pol III promoter

For expressing ECX/MUT sequences under U6 promoter we cloned ~400 ECX/ECX_MUT sequence pairs into pLKO.1 plasmid using AgeI and EcoRI sites, and transduced the MDA-MB-231 by lentiviral transduction as described above.

MDA-MB-231 cells expressing REX or RIX sequences under pol III promoter

For expressing REX1-8/RIX1-3 sequences under U6 promoter we cloned oligos in Table S3 into pLKO.1 plasmid using AgeI and EcoRI sites, and transduced the MDA-MB-231 by lentiviral transduction as described above.

MDA-MB-231 cells expressing REX or RIX sequences under pol II promoter

For cloning REX1, REX5 and RIX1 sequences under the CMV promoter, we first cloned the ribozyme-small RNA-ribozyme (HH/HDV) cassette[18] into BdLV_Puro_mCherry using PacI and MluI site. We then digested the vector using AsiSI and cloned gblocks containing the sequence of interest (Table S4) using Gibson assembly. Plasmids were delivered to MDA-MB-231 by lentiviral transduction as previously described.

RT-qPCR for REX/RIX expression

3.5 μl of isolated RNA was polyA tailed by adding 0.5ul 10X polyA polymerase buffer, 0.5ul 10mM ATP, 0.25ul polyA polymerase (NEB),0.25 μl H2O and incubating at 37°C for 10 min. 2.5 μl polyA tailed RNA was then reverse transcribed by adding 0.25ul 10mM dNTPs, 0.1ul 100uM dT T7 primer, 5X RT buffer, 0.15 μl RNAseOUT, 0.25 μl Maxima H Minus RT and 0.75 H2O by incubating

at 50C for 15 min followed by 85C for 5 min. QPCR was done using PerfeCTa SYBR Green SuperMix, T7 primer, and miRNA specific primer as listed on Table S5. Mir16 primer was used as an endogenous control. To select for EC enriched REX smRNAs, we used both the abundance and enrichment of small RNAs in the EV and CM fractions as a selection criteria. The criteria for Ct and log-fold EV and CM enrichment values were set to be one standard deviation below and above the respective averages of these values for the RIX sequences.

Generation of smRNA libraries

ECX/MUT and REX/RIX oligo pools were ordered from Twist Biosciences. Both oligo pools were separately cloned into pLKO.1-puro plasmid using AgeI and EcorI sites and were transformed into MegaX electrocompetent cells with about 1000X coverage. The smRNA libraries were then transfected to MDA-MB-231 cells using lentivirus as described previously. We maintained a 1000x coverage through the transduction process.

RNA isolation from conditioned media (CM) and extracellular vesicles (EV)

MDA cells were seeded in 10 cm or 15 cm plates. The next day the media was removed, and cells were washed with 1X PBS. Cells were then incubated in media prepared with exosome depleted FBS (cat# A2720801) in standard cell culture conditions for 48 h. After 48 h, media was collected and spun down at 500g and passed through 0.4 μm to remove any cells. For RNA isolation from conditioned media (CM), we took 1 mL of cell free media and performed RNA isolation using zymo research *Quick*-cfRNA Serum & Plasma Kit (cat# R1059).

The rest of the media was used for exosome isolation. We took advantage of an EV enrichment method using polyethylene glycol (PEG) as outlined in patent# EP2839278B1.[37] by adding Polyethylene Glycol 10000 (PEG, HR2-607) to 10% final and overnight incubation at 4C. The next day PEG/media mixture was spun down at 3000 g at 4C for 1 h. We then removed the supernatant and proceeded to Zymo Research Quick-RNA Microprep Kit (cat#R1051) for RNA isolation from the EV pellets observed at the bottom of the tube. To confirm the efficacy of our EV isolation through the PEG precipitation method, we present a western blot image of CD81, a well-established exosomal marker, detected in EVs isolated from MDA-MB-231 conditioned media (Figure S5). The blot was stained with 1:2000 anti CD81 and 1:10000 IRDye 800CW Goat anti-Mouse IgG Secondary Antibody and visualized using Licor Odyssey XF.

To delve further into whether the RNA captured in the CM fraction is actively secreted through mechanisms involving lipoprotein complexes, rather than being a result of passive mechanisms like cell death, we conducted a repeated experiment as shown in Figure 2A. This time, we divided the media into two conditions: one with RNAse treatment and one without RNAse treatment. We then extracted RNA from the conditioned media and performed small RNA sequencing. Our analysis revealed that there were no significant differences in RNA sequences (correlation coefficient, R = 0.81) between the two treatment conditions. This observation suggests that the RNA sequences in our ECX sequences are mostly secreted through EVs and are thus protected by the RNAse treatment, as they remain relatively unaffected by the enzymatic degradation.

RNA isolation from cells

Total RNA for RNA-seq and RT-qPCR was isolated using the Zymo Research Quick-RNA Microprep Kit (cat#R1051) with in-column DNase treatment per the manufacturer's protocol.

ExoCLIP

ExoCLIP of flag tagged RBM24, HNRNPA2, HNRNPB1 MDA-MB-231 cells was done by seeding 12M cells divided in four 15 cm cell culture plates for each cell line in DMEM media as described above. After 24 h, the media was changed to DMEM with exosome free FBS. 48 h after the media change, the conditioned media was collected and transferred to 50 mL falcon tubes and spun once at 500g and once at 2000g for 10 min at 4C to clarify the media from any cells. Clarified media was then transferred to 15 cm plates for crosslinking at 200 mJ/cm^2 254 nm UV. After the first UV exposure we swirled the media and repeated the crosslinking step for a second time. Crosslinked clarified media was transferred to the centricon plus-70 filter 10K MWCO (millipore sigma UFC701008) and concentrated according to the manufacturer's protocol.

To the concentrated media we added protease inhibitor, SuperaseIN, EDTA, 1M Tris-HCl pH 7.5, and anti-flag magnetic beads (CAT# A36797) and incubated with rotation for 20 h at 4°C. Beads were magnetized and washed sequentially with cold low salt wash buffer, high salt wash buffer and PNK buffer two times each. This was followed by a PNK mediated dephosphorylation step (2.5ul 10X PNK buffer, 2ul 10X T4 PNK (10unit/ul), 0.5ul SuperaseIN, 20ul H2O) for 20 min at 37C and sequential washes with PNK buffer and high salt wash buffer.

The de phosphorylated RNA-protein complexes were then poly A tailed using yeast PAP, PAP buffer, ATP and SuperaseIn (Jena 600U/ul) at 22 for 5 min. The poly A tailed RNA-Protein complex was then labeled by N3-dUTP, and yeast PAP, PAP buffer and SuperaseIn at 37C for 20 min. Beads were then washed by high salt wash buffer and PBS. The N3-labeled smRNA was stained with 1mM 800cw DBCO at 22C for 30 min. Beads were magnetized and washed with high salt HITS-CLIP WB and PNK buffer respectively, and then resuspended in 20ul of 1X NuPAGE loading buffer +50mM DTT final concentration diluted in PNK buffer and heated at 75C for 10 min. Beads were placed on the magnet for elution. The eluted RNA protein complexes were then frozen in −80 and later used for WB analysis as described below.

Low salt wash buffer

1X PBS (TC grade, no Mg++, no Ca++)
1% IGEPAL CA-630.
High Salt Wash Buffer:
5X PBS (TC grade, no Mg++, no Ca++)
1% IGEPAL CA-630.
1X PNK Buffer:
50mM Tris-Cl pH 7.4.
10mM MgCl2.
1% IGEPAL CA-630.

Western Blotting

Eluted RNA-protein complexes from above were run on SDS-PAGE using 4–12% Bis-Tris NuPAGE gels and transferred to protran BA-85 nitrocellulose membrane. The membrane was briefly rinsed in PBS and placed in a sheet protector and imaged with a Licor Odessey instrument.

Protein K digest and RNA capture

The RNA-protein complexes imaged as described above appeared as a diffused signal with a modal size of ~15-20kDa above the expected MW of the protein of interest. Average MW of 21 nt long RNA is ~7kDa. Poly(A) tail ~20nt (~6.5kDa), therefore the position of the protein-RNA complex that will generate CLIP tags longer than 20nt is ~14kDa above the expected MW of the protein. HNRNPA2-Flag and HNRNPB1-Flag run at 38 and 39 kDa respectively and RBM24-Flag runs at ~28. Therefore, we cut between 55 and 85 kDa for HNRNPA2 and HNRNPB1 lanes and 39–70 kDa for RBM24 lane. The MDA only (no flag) lane was cut from 39 to 85 kDa.

The cut membranes were each transferred to a 1.5 mL Eppendorf tube and treated with 12.5 μl Proteinase K in 200 μl Proteinase K digestion buffer at 55C for 45 min. The samples were then quickly spun down and the 200 μl of supernatant was transferred to a clean Eppendorf tube. Samples were then adjusted for salt by adding 19 μl 5M NaCl and 11 μl H2O per 200 μl sample.

To capture the RNA, we used 30 μl Oligo d(T)25 dynabeads (Invitrogen cat#61002) per IP. Beads were washed 2X with Proteinase K buffer before use. We transferred ~200ul salt-adjusted samples to the beads and incubated at 25C at 300 RPM for 20 min with occasional shaking of 1350 RPM. We then washed the samples/beads 2X with cold high salt wash buffer and 2X with PBS, magnetized and removed the supernatant. RNA was eluted by incubating the beads in 8 μl TE elution buffer at 50C for 5 min. Beads were magnetized and 7.5 μl of eluted RNA was transferred to clean PCR tubes.

Small RNA library preparation

Small RNA library preparation for samples taken from exoCLIP was done using Takara Bio SMARTer smRNA-Seq Kit (cat# 635029) with a few modifications. Since our RNA was already poly-A tailed, we skipped this step in the protocol and moved to the cDNA synthesis. We also wanted to incorporate UMI in our cDNA, so we added 2.5ul smRNA mix 1 and 1ul of 10uM dT-UMI RT primer to our 7.5 μl poly A-tailed smRNA and incubated at 75C for 3 min and then placed on ice for 5 min. We then performed reverse transcription as described in the kit's protocol. In the PCR step we also added a 2 μl, 10 μM Universal reverse primer (P7) to the PCR mix and added the 78 μl mix to each cDNA sample. We then added the 2 μl index forward primer to each sample and incubated as described in the protocol. We purified the PCR product using Zymo Research select-a-size MagBead (cat#D4084-50). ExoCLIP sequencing primers and barcodes are listed under Table S6.

Library preparation for RNA isolated from ECX/MUT or REX/RIX transduced MDA-MB-231 cells and small RNA library from HNRNPA2B1 KD and RBM24 KD MDA-MB-231 cells were prepared using an in-house small RNA library preparation. 7.5 μl RNA was polyadenylated using 1ul NEB 10X polyA pol buffer, 1 μl 10 mM ATP, 0.25 μl RiboLock (40 u/ul), 0.25 μl E. coli PolyA pol. 5 u/μL (NEB), and incubated at 16C for 5 min, and then put on ice for maximum 5 min before proceeding to cDNA synthesis. We added 1 μl of RT primer, incubated at 72C for 3 min before putting on ice. We then prepared RT mix on ice using 2 μl 5X RT buffer with DTT (Thermo), 1 μl 10 mM dNTP, 4 μl 5M Betaine, 1 μl Maxima H- RT 200 u/μL (Thermo), 0.25 μl RiboLock 40 u/μL (Thermo), 1 μl 10 μM TSO-UMI primer. We incubated the RT mix at 42C for 30 min and then at 85C for 5 min. The cDNA amplification was carried by using 19 μl cDNA from previous step, 20 μl 5X Phusion HF buffer (Thermo), 2 μl 10 mM dNTP, 2 μl 12 μM Takara Fwd PCR primer, 2 μl 12 μM 12 μM Takara Rev PCR primer, 1 μl Phusion HS II pol. 2 u/μL (Thermo), and 48 μl H2O. We then ran a PCR reaction for cDNA amplification as follows: 30 s @ 98C - [10 s @ 98C - 10 s @ 65C - 5 s @ 72C]xN cycles (N determined by performing a qPCR). RT, TSO and Takara primers are listed under Table S7. Sample barcodes and indices are listed under Tables S8 and S9.

PCR reaction was purified through an MN NucleoSpin Gel & PCR Cleanup column (cat#740609) and eluted in 30 μL of water. We ran samples on a 8% TBE gel for 35 min at 180V and stained the gel with 1X GelGreen in 1X TBE for 2 min then imaged under Blue light. We cut the fragment of interest (150–200 bp) and placed the gel slices in a 0.5 mL tube with a hole pierced by a 18G needle. Spun the tube in a 1.5 mL tube until the gel was passed through the hole. We then added 400 μL of the DNA gel extraction buffer (10 mM Tris pH 8, 300 mM NaCl, 1 mM EDTA) to the gel, vortexed and froze on dry ice for 30 min, and then thawed overnight on a rotator. Next day we transferred the gel slurry to a Costar filter spin-column and spun at maximum speed until all liquid has passed through. We added

1.5 μL of GlycoBlue and 500 μL of isopropanol to the DNA solution, and put in −80C for 1 h, then spun at 4C for 30 min, air dried for 10 min, and resuspended and incubated in 10 μL of 10 mM Tris pH 8 for 10 min.

Sequencing and analysis

Libraries were quantified using Qubit HS dsDNA kit, and also ran on an Agilent bioanalyzer HS DNA Chip or HSD1000 tapestation. All libraries were sequenced as SE65 runs on Illumina Hiseq 4000 at UCSF Center for Advanced Technologies. Reference sequences were collated as fasta files (for ECX and MUT sequences, and RIX and REX sequences separately).

UMI-tools[38] (v1.0) and cutadapt[39] (v3.5) were used to extract UMIs and remove linker sequences. BWA[40] (v0.7.17) was used to align reads and then duplicates were removed using the extracted UMIs. Reads mapping to each sequence in the bam file was then counted and DESeq2 (v1.24) was then used to normalize and compare the extracellular fractions to the intracellular fraction and log2 fold-change were reported for each group of sequences.

For the analysis of ECX/MUT experiment in Figure 2A, we included 55 ECX RNAs and 55 matched MUT RNAs. For this analysis, we required the RNA to be present in a third of samples in both EV and CM fractions so that the logFC values were meaningful.

HITS-CLIP

HITS-CLIP for endogenous HNRNPA2B1 was done as described by (Licatalosi et al., 2008)[41] with the modifications previously used for YBX1 small RNA CLIP (Goodarzi et al., 2015).[24] MDA-MB-231 cells were UV-crosslinked at 400 mJ/cm^2 before cell lysis. Samples with and without RNase treatment were immunoprecipitated with an anti-HNRNPA2B1 antibody (Thermo, PA5-34939) for protein-RNA complexes. RNase treatment was as follows: RNase A (Affymetrix 70194Z, 9,063 units/mg, 4.89 mg/mL); low RNase was 1:2500 and high RNase was 1:50 dilutions, the no RNase was 0 RNase.

Polyphosphatase (Lucigen) was incubated with smRNA samples before ligation and PCR amplification with primers described by (Goodarzi et al., 2015).[24] Constructed libraries were sequenced on the Illumina HiSeq2000 at the Rockefeller University Genomics Center. The resulting library was then analyzed using the CLIP toolkit (CTK).[30]

Data Acquisition

To train our predictive models, we sourced small-RNA sequences data for intracellular and exosome-specific predictions from three reliable sources: Goodarzi et al. (GSE114366),[7] Extracellular RNA Communication Consortium Atlas (exRNA Atlas), and The Cancer Genome Atlas (TCGA). We first used the GSE114366 data, which was generated to investigate the roles of intracellular and extracellular small-RNAs in breast cancer. We extracted small-RNA-seq data of intracellular small-RNAs (IC) and small-RNAs present in extracellular vesicles (EV) from 8 different breast cancer cell lines. Although the small-RNA selection and secretion machinery may differ among different cell types and states, we assumed that there are some general and common mechanisms that exist in cells. Therefore, we merged all of the IC data, regardless of the cell line, and imported 30,093,690 IC-resided small-RNA sequences to our IC dataset. We also integrated all the EV data and collected 6,127,883 EV-resided small-RNA sequences to our EV dataset. Second, we imported 67,511,039 EV-resided small-RNA sequences from the exRNA Atlas, which were extracted from the serum part of blood cells of 12 samples. Third, we collected 1,8488,703 IC-resided miRNA sequences from the TCGA dataset which were extracted from normal cells of different tissues across the body. We selected miRNA-seq data and not RNA-seq data because our main focus in this research is to investigate the selection and secretion processes of small-RNAs inside a cell. Notably, to avoid data leakage due to sequence similarity, we filtered out redundant (i.e., highly similar) sequences from the aggregated dataset. The table below presents information regarding the total number of samples utilized from each dataset, as well as the percentage of samples held out from each dataset.

Data preprocessing

After integrating data from three distinct sources, we performed several preprocessing steps to clean and organize the data. We first removed sequences that were present in both the IC and EV datasets, assuming that they belonged to the EV, as exported small-RNA sequences can also exist intracellularly. We then eliminated sequences that were less than 18 nts or more than 50 nts, as our focus was on small-RNAs. We also removed any sequences that contained "N" in their primary sequence in order to decrease ambiguity in our dataset. We eliminated duplicated sequences and sequences that were a substring of a bigger one. For example, if we had both ACGU and UACGU sequences in our dataset, we removed the former one. To further clean the data, we used the MEME-Suite’s dust tool to mask and delete sequences that carried low-complex regions. The dust tool helps to identify and remove any non-informative regions in the sequences such as repetitive regions. After all of these preprocessing steps, we had 33,083 unique EV small-RNAs and 1,318,795 unique IC small-RNAs that were ready to be used for predictive model training.

Feature generation

We derived multiple features from IC and EV small-RNA primary sequences to train the classifiers, utilizing the ViennaRNA package to predict RNA secondary structures and free energies for each RNA within our dataset. Two distinct secondary structure representations were generated using ViennaRNA. The first format, created using the RNAfold module, employed a dot-bracket notation. In this notation, nucleotides were represented as either single-stranded, indicated by a dot (.), or double-stranded, denoted by open and closed brackets (e.g., "(" or ")").

The second representation format, referred to as the bulge-graph notation, was created using the forgi module. This representation categorized RNA secondary structures into five distinct types: five-prime (f), three-prime (t), stem (s), interior loop (i), multiloop segment (m), and hairpin loop (h).

To expand the representation of these sequences, we developed an 8-term notation that encapsulated information from both the primary sequence and secondary structures. In this notation, each sequence was represented using the characters {A, C, T, G, a, c, t, g}, where uppercase characters indicated double-stranded nucleotides characterized by bracket secondary structures, and lowercase characters represented single-stranded nucleotides indicating dot secondary structures.

In summary, for each small RNA in our IC and EV dataset, we had four distinct sequence representations: the primary sequence, the dot-bracket secondary sequence, the bulge-graph secondary sequence, and the nucleotide-based secondary sequence. Additionally, we obtained the predicted free energy for each RNA.

These sequences were further analyzed by extracting K-mers (K = 1–4, 5, 7) from both the primary and secondary sequences mentioned above. K-mers, or k-grams, represent substrings of length k within any given sequence. For example, for 3-mers, combinations such as " … ", ".(", ".(.", "(.", "(.)", ".)", ".).", and "())" were generated for the dot-bracket representation.

To ensure uniformity in our dataset, we normalized the features, including K-mer frequencies and free energy, based on the length of each sequence. Additionally, we included the length of the sequence as a feature.

Given that the majority of our sequences had lengths less than 50 base pairs (bp), sequences exceeding 50 bp in length were truncated, and smaller sequences were padded to achieve a fixed length. This preprocessing step ensured that our data was consistent and ready for input into our model.

Predictive models

The predictive models examined in this research are divided into two categories: classical machine learning methods and deep learning methods. Classical methods trained/tested in this research include support vector machines (SVMs) and random forests (RFs). On the other hand, deep learning methods include models which are inspired by convolutional neural networks (CNNs) and recurrent neural networks (RNNs). It should be noted that due to the limitations of machine learning models (SVMs and RFs) in handling sequence-based data, all sequence-typed features were removed from the final design matrix for the machine learning experiments.

Support Vector Machines

Support Vector Machines (SVMs) are a family of supervised machine learning algorithms that are commonly used for linear and non-linear classification tasks, as well as for regression tasks. In this study, we conducted an ablation study to evaluate the effectiveness of different input feature spaces and kernel types for SVMs. Specifically, we defined six different training scenarios, which varied in terms of the input feature space and kernel type used.

To evaluate the effectiveness of non-Kmer features, we used two different feature sets: one that included all extracted features, and another that included only the K-mers extracted from the primary sequences. Additionally, we tested both linear and Radial Basis Function (RBF) kernels for the SVMs. Furthermore, due to the inherent class imbalance issue existing in the preprocessed dataset, we tried to mitigate this issue by using weighted SVM by weighting the class parameters inversely proportional to their sample frequencies.

Random Forest

In this study, we employed Random Forest, a powerful tree-based machine learning algorithm, to train on the preprocessed dataset. This choice was made due to the algorithm's ability to handle large feature sets and its robustness to overfitting compared to other existing machine learning models. Similar to the experiments conducted with Support Vector Machines (SVMs), we evaluated the effectiveness of both weighted and unweighted Random Forest models. Additionally, we investigated the impact of different tree population sizes on the performance of the algorithm. Specifically, we tested tree population sizes of 50 and 200 while keeping the tree-depth fixed at 20.

ExoGRU

ExoGRU, as the name suggests, consists of multiple GRU units stacked on top of each other. GRUs introduced a simpler alternative compared to LSTMs. They are able to capture relatively long-term dependencies by utilizing gates in order to control the information flow.

A GRU unit computes the hidden state at time step t as follows:

$$z_t = sigmoid(W_z x_t + U_z h_{t-1} + b_z)$$

$$r_t = sigmoid(W_r x_t + U_r h_{t-1} + b_r)$$

$$h'_t = tanh(W_h x_t + U_h(r_t * h_{t-1}) + b_h)$$

CellPress
OPEN ACCESS

$$h_t = (1 - z_t)h_{t-1} + z_t h'_t$$

Where x_t is the input at time step t, h_{t-1} is the previous hidden state, W_z, U_z, W_r, U_r, W_h, U_h are the weight matrices, b_z, b_r, b_h are the bias terms and sigmoid and tanh are non-linear activation functions. The update gate z_t and reset gate r_t are used to control the flow of information into the hidden state h_t, allowing the network to better handle long-term dependencies.

ExoLSTM

ExoLSTM is also another network we employed in this study. The architecture consists of multiple LSTM units stacked on top of each other. Long Short-Term Memory (LSTM) units are a type of recurrent neural network (RNN) that uses a memory cell to store information over a longer period of time. The memory cell is controlled by gates that determine when to store, update, or discard information in the cell.

An LSTM unit computes the hidden state at time step t as follows:

$$i_t = sigmoid(W_i x_t + U_i h_{t-1} + b_i)$$

$$f_t = sigmoid(W_f x_t + U_f h_{t-1} + b_f)$$

$$o_t = sigmoid(W_o x_t + U_o h_{t-1} + b_o)$$

$$c_t = f_t * c_{t-1} + i_t * tanh(W_c x_t + U_c h_{t-1} + b_c)$$

$$h_t = o_t * tanh(c_t)$$

Where x_t is the input at time step t, h_{t-1} is the previous hidden state, c_{t-1} is the previous memory cell state, W_i, U_i, W_f, U_f, W_o, U_o, W_c, U_c are the weight matrices, b_i, b_f, b_o, b_c are the bias terms, and sigmoid and tanh are non-linear activation functions. The input gate i_t, forget gate f_t, output gate o_t and cell state c_t are used to control the flow of information into the hidden state h_t, allowing the network to better handle long-term dependencies.

ExoCNN

ExoCNN is a variant of convolutional neural networks (CNNs) designed to generate predictions from sequences. The architecture of ExoCNN is composed of several layers, including convolution, pooling and fully connected layers, each of which contains tunable weights and biases. One key aspect of the ExoCNN architecture is the use of "conv blocks" as firstly defined in VGG[42] which are composed of multiple consecutive convolution layers followed by a max-pooling operation. In the max-pooling operation, the maximum value is computed for each window of size 2 in the "conv block"'s output matrix, this helps to summarize spatial information into the output while retaining the spatial information. Following the "conv blocks" and max-pooling operations, the output of the last max-pooling operation is flattened and fed to a classifier head with 2-layer fully connected neural network. Similar to the convolution layers, rectified linear activation functions are used in the head. The number of neurons in the hidden layers of ExoCNN's classification head are 1024 and 128 respectively. Finally, the output of the last layer is passed through a sigmoid function which generates a (secretion) probability for the input sequence.

Model training

As shown in Table 1, the sample frequency of the IC class is significantly higher than the EV class, which results in a common problem in machine learning known as class imbalance. To address this issue, we downsampled the IC dataset to balance the class frequencies. Additionally, we employed the weighted cross-entropy (WCCE) loss function for training our ExoCNN model in which each class weight is inversely proportional to its sample frequency. The original EV dataset and the downsampled IC dataset were used to train our predictive models. To assess the performance of deep learning models, we performed stratified train/validation/test split with proportions of 0.8, 0.1, and 0.1 on our preprocessed dataset.

We used the Adam optimizer with a learning rate of 0.001 for 100 epochs for all DL models. We employed a batch size of 128 during training. To prevent overfitting, we employed early stopping and learning rate decay techniques during the training process. To initialize the weights of each layer in the network, we used the Xavier initializer. Additionally, we used L1 and L2 regularization techniques with a lambda value of 1e−6 to further prevent overfitting.

Motif discovery and enrichment

ExoGRU works within a binary classification framework, each sequence receiving a secretion probability computed from the sigmoid-transformed output of the network's predictions. As demonstrated in Niculescu-Mizil and Caruana et al.,[43] neural networks trained for binary classification tasks typically yield well-calibrated probabilities, implying that the probabilities generated by ExoGRU serve as reliable estimations of the confidence we place in the model's predictions. Therefore, after training the network with small-RNA sequences, two sets of sequences were identified that the model was highly confident about being secreted or not. Sequences of extracellular vesicles (ECs) are assigned as ECX if the calculated probability exceeds 0.95. Similarly, intracellular (IC) sequences are designated as ICX if the associated probability falls below 0.05 (Table 2).

In order to find motifs more accurately, we removed highly similar sequences from the ECX and ICX sets using the MEME-Suite's purge tool. To find the optimal similarity score threshold, we experimented with different thresholds and checked the number of sequences and removed ones for each threshold. Finally, we used a similarity score threshold of 50. The extreme sequences (ECX and ICX) were clustered based on edit distance and cosine distance. We found motifs based on both unclustered and clustered ECX and ICX, but the results were the same, so we eliminated the clustering step from the analysis pipeline. To perform an exhaustive motif search, we used several motif finders and tested various configurations of the tools. Three motif finding tools were able to discover motifs in the small-RNA sequences: MEME, Homer, and FIRE. We used these tools with three different input sets: ECX only, ECX vs. ICX, and ECX vs. randomly generated sequences that preserved di-nucleotide frequency. Using these three motif finding tools, three different configurations, and several parameter tuning, we found 10 motifs that were enriched in the ECX sequences. These motifs were related to previously known RNA-binding proteins that are involved in the secretion machinery, and were presented in Figure 4.

With the discovery of the ECX and ICX sequences and the corresponding motifs, we continued our research by identifying secretion-related RNA-binding proteins in two distinct ways. First, we compared the discovered motifs with already known ones in the literature and databases. This allowed us to identify any previously known motifs that were enriched in the ECX sequences and related to known RNA-binding proteins involved in the secretion machinery. Second, we analyzed the eCLIP-seq data of the ENCODE project to identify binding sites of human's RNA-binding proteins. This allowed us to identify any potential RNA-binding proteins that may be involved in the secretion of small-RNAs based on their binding sites in the ECX and ICX sequences.

Motif comparison

We compared the 10 discovered motifs with known motifs of RNA-binding proteins to detect the proteins that are highly likely to bind to each motif and participate in the secretion machinery. To do this, we used three databases of Ray2013, RBPDB, and ATtRACT, and the MEME-Suite's Tomtom tool to find RNA-binding proteins (RBPs) that significantly bind to our discovered EV-enriched motifs. This motif comparison process gave us 7 proteins that are highly likely to bind to our secretion-related motifs, as shown in Figure 4. As previously mentioned, two of these proteins have already been verified to be involved in the secretion machinery. This comparison process helps us to identify potential players in the secretion process and further investigate them.

RBP binding sites analysis

We aimed to identify RNA binding proteins (RBPs) in the ENCODE database that may have greater interactions with extreme EV sequences, as opposed to IC sequences. Our hypothesis is that these proteins may play a role in the secretion machinery. We filtered out proteins that did not have signals (bigWig file) or peaks (BED file) as their output type and that were not based on the GRCh38 reference genome. This resulted in a final selection of approximately 150 proteins.

To begin, we determined the maximum signal value at each nucleotide position for a specific protein if we have multiple experiments (bigWig files). Next, we extracted signal values for nucleotide positions that overlapped with peak regions, separately for IC and extreme EV sequences. We then used the Mann-Whitney statistical test to compare these two sets of signal values and calculate a p value to determine if the EV signals were significantly greater than the IC ones.

To obtain comparable signal intensity values and gain a deeper understanding of the interactions between EV extreme sequences and RBPs, we evaluated various scoring methods. In our initial analysis, we obtained signal values (scores) for EV sequences and assigned zero values to regions that did not overlap with peak regions for a specific protein. We also applied this method to IC sequences. This resulted in many zero values in our scores, and the Mann-Whitney test showed a significant sensitivity to the mean in these scenarios.

To address this issue, we modified our approach. Instead of using all peak regions, we applied it to the union of IC and extreme EV regions. This eliminated many zero values from the scores and allowed us to better understand the natural behavior of RBPs. We found that they tend to bind to EV extreme sequences with high signal values and to IC extreme sequences with moderate signal values on average. We then used the Benjamini-Hochberg (BH) method to adjust our p values and identified proteins with adjusted p values less than 0.05 as being involved in the EV secretion machinery.

After analyzing the interactions between different proteins and RNA sequences, we also took an intra-protein approach to the problem. To obtain information within each sample (IC vs. EV), we used extreme sequences of both IC and EV groups with a secretion probability greater than 0.9 that overlapped with peaked regions. We extracted several features including: the number of EV and IC extreme sequences overlapping with the peaked regions, the total length of each overlapping extremes with peaked regions, the total

CellPress
OPEN ACCESS

sum of the signal values for each overlapping extremes, and the mean value of signals for each of the extremes. With this data, we could assess the robustness and reliability of our results as a sanity check and also identify any potential outliers related to the secretion machinery. To do this, we used median absolute deviation (MAD), Z-test, and Percentile rank.

Model Interpretability

A number of strategies have been developed in recent years to help interpret neural network models. For simpler models, such as DeepBind, the convolutional kernels themselves were used to represent features captured by the model.[15] However, for the more complex architectures, including deeper CNNs or RNNs, that are trained on heterogeneous data, customized feature importance analyses are employed. Most famously, DeepLIFT uses a variation of integrated gradient to select those partial sequences across inputs that are most important for the model's prediction and then performs a motif discovery in them, using TF-MoDIsco.[29] In other words, DeepLIFT massively reduces the space in which motif discovery is performed by removing the sequences and parts of sequences that are not informative for the model. Motif discovery can then be effectively performed to identify the features that the model is learning in these sequences. In our case, however, since the sequences are already short for small RNAs, the DeepLIFT scoring is not needed, and we can directly perform motif discovery. However, motif discovery is only performed on sequences that the model is confidently classifying (i.e., ECX). In other words, for these sequences, the model has learned strong features that enable it to make a correct and confident prediction. This approach is very different from performing motif discovery on the initial labels, both in theory and practice. By focusing on the ECX sequences, we are strongly enriching the signal from these sequence features. This is crucial because RNA secretion is a complex process with multiple pathways and many players involved.

To investigate ExoGRU in a more fine-grained way, we extracted approximately 5,000 IC and EC sequences that contain a specific motif associated with RBM24, namely GAGUC. These selected sequences were collectively labeled as "gaguc-intact" and served as the focus of our investigation. We employed DeepLIFT tool to assess the significance of different sequence regions in influencing the model's predictions.

Furthermore, we conducted a masking and ablation procedure on the gaguc-intact sequences, called them gaguc-masked and gaguc-removed sequences to show that the model relies on this motif for its prediction. These sets of modified sequences were then used as input to the model, enabling us to examine any changes in the model's predictions. Similarly, We applied the same procedure on the other two motifs (CCUGGC, and [ACU]AG[GU][GU]) as well.

Cell Genomics, Volume *4*

Supplemental information

Revealing the grammar of small RNA secretion using interpretable machine learning

Bahar Zirak, Mohsen Naghipourfar, Ali Saberi, Delaram Pouyabahar, Amirhossein Zarezadeh, Lixi Luo, Lisa Fish, Doowon Huh, Albertas Navickas, Ali Sharifi-Zarchi, and Hani Goodarzi

Supplementary Figures

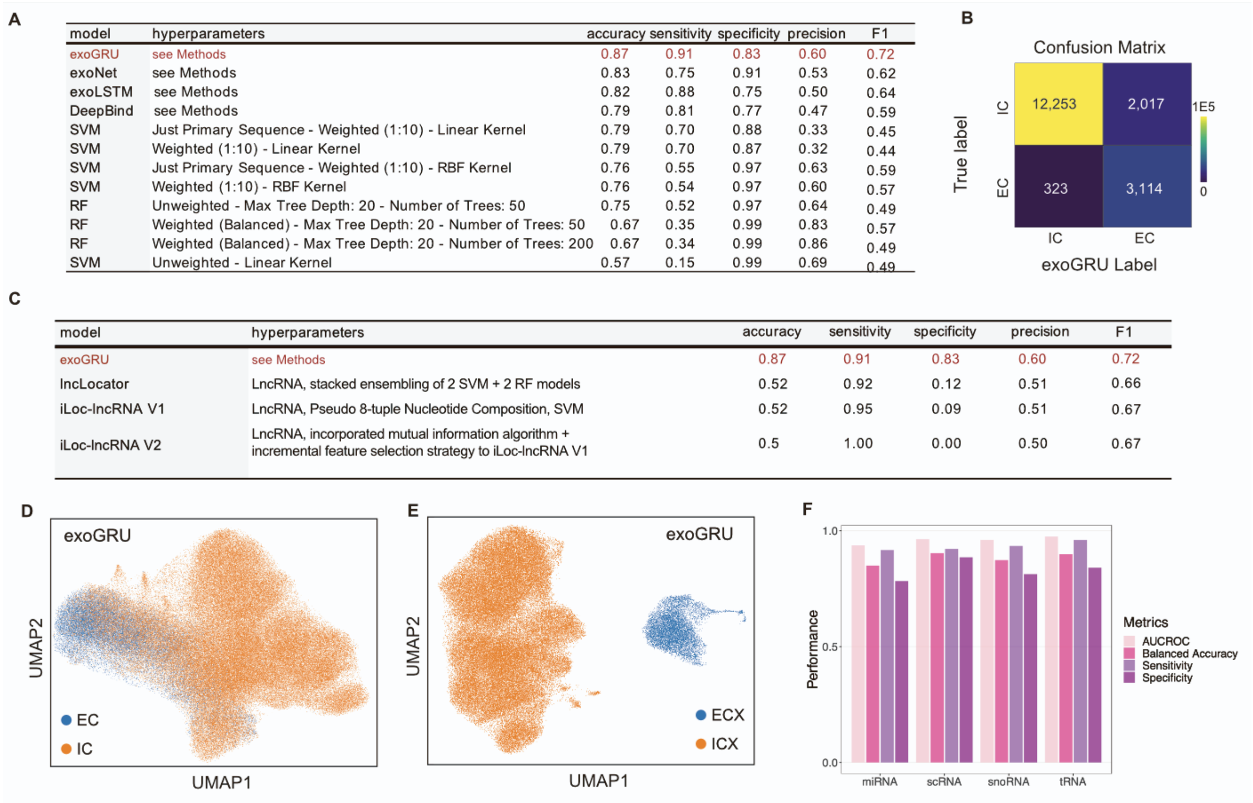
A

model	hyperparameters	accuracy	sensitivity	specificity	precision	F1
exoGRU	see Methods	0.87	0.91	0.83	0.60	0.72
exoNet	see Methods	0.83	0.75	0.91	0.53	0.62
exoLSTM	see Methods	0.82	0.88	0.75	0.50	0.64
DeepBind	see Methods	0.79	0.81	0.77	0.47	0.59
SVM	Just Primary Sequence - Weighted (1:10) - Linear Kernel	0.79	0.70	0.88	0.33	0.45
SVM	Weighted (1:10) - Linear Kernel	0.79	0.70	0.87	0.32	0.44
SVM	Just Primary Sequence - Weighted (1:10) - RBF Kernel	0.76	0.55	0.97	0.63	0.59
SVM	Weighted (1:10) - RBF Kernel	0.76	0.54	0.97	0.60	0.57
RF	Unweighted - Max Tree Depth: 20 - Number of Trees: 50	0.75	0.52	0.97	0.64	0.49
RF	Weighted (Balanced) - Max Tree Depth: 20 - Number of Trees: 50	0.67	0.35	0.99	0.83	0.57
RF	Weighted (Balanced) - Max Tree Depth: 20 - Number of Trees: 200	0.67	0.34	0.99	0.86	0.49
SVM	Unweighted - Linear Kernel	0.57	0.15	0.99	0.69	0.49

C

model	hyperparameters	accuracy	sensitivity	specificity	precision	F1
exoGRU	see Methods	0.87	0.91	0.83	0.60	0.72
lncLocator	LncRNA, stacked ensembling of 2 SVM + 2 RF models	0.52	0.92	0.12	0.51	0.66
iLoc-lncRNA V1	LncRNA, Pseudo 8-tuple Nucleotide Composition, SVM	0.52	0.95	0.09	0.51	0.67
iLoc-lncRNA V2	LncRNA, incorporated mutual information algorithm + incremental feature selection strategy to iLoc-lncRNA V1	0.5	1.00	0.00	0.50	0.67

Supplemental Figure 1. ExoGRU confusion matrix and embedding visualization, related to figure 1
A) Table compares various quality metrics of ExoGRU's performance against different learning models tested**.** In most categories ExoGRU performs better than all the other models. **B)** The confusion matrix illustrates the comparison between ExoGRU-predicted EC and IC labels and their corresponding true labels from the datasets. A total of 3437 sequences with a true EC label were tested, of which approximately 90% were correctly identified as EC by ExoGRU. Similarly, 14270 sequences with a true IC label were tested, with ExoGRU correctly labeling approximately 86% of them as EC. **C)** Comparison of ExoGRU model with other existing models for prediction of RNA subcellular localization. Quality metrics are also listed for ExoGRU and all other existing models **D)** UMAP projection was used to visualize the 64-dimensional embedding of EC vs IC. EC labeled sequences are those with > 0.5 secretion probability and IC sequences have < 0.5 secretion probability as predicted by ExoGRU. **E)** UMAP projection shows the 64-dimensional embedding of high confidence ECX and ICX calls. ECX labeled sequences are those with > 0.95 secretion probability and ICX sequences are those with < 0.05 secretion probability as predicted by ExoGRU. **F)** Comprehensive analysis of ExoGRU's performance in predicting subcellular localization across various small RNA subtypes including miRNA, scRNA, snoRNA, tRNA.

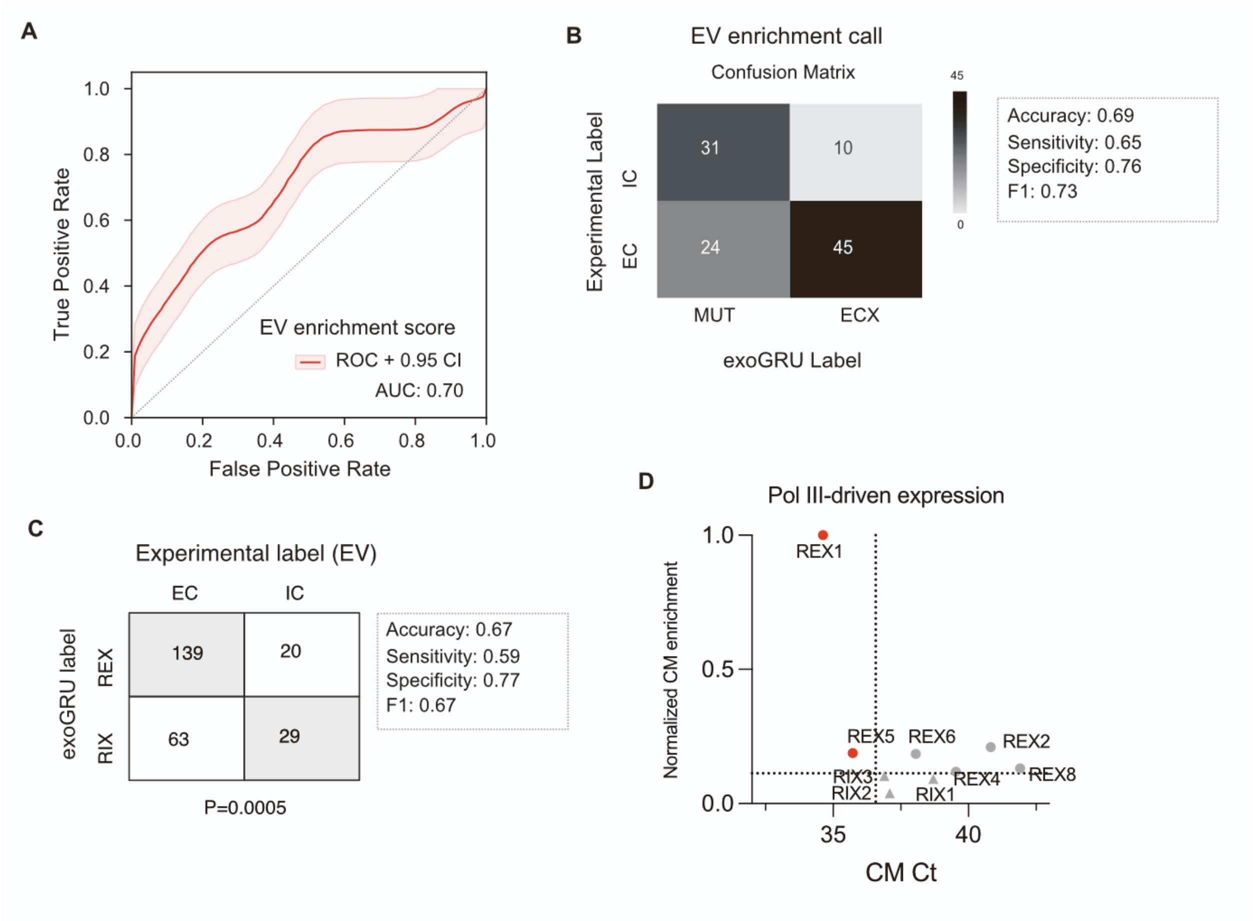

Supplemental Figure 2. ROC curve and confusion matrix for ExoGRU predictions and its experimental validations, related to figure 2. A) ROC curve generated using ECX and MUT experimental EV enrichment score and ExoGRU's localization predictions to measure the association between the experimental vs ExoGRU labels at every classification threshold. The smoothened ROC curve was generated by performing 1000 bootstraps. **B)** Using ROC curve analysis, EC and IC labels were assigned to sequences from conditioned media (EV enrichment) using a specificity threshold of 0.75. These experimental labels were subsequently employed to construct a confusion matrix for the classification of ECX and MUT sequences. Performance metrics are provided for this classification. **C)** The presented contingency table illustrates the experimental distribution of ExoGRU generated REX and RIX sequences in EV. The ExoGRU class predictions for these synthetic sequences achieved an accuracy of 67%, with 59% sensitivity and 77% specificity. A χ^2 test was applied to calculate a p-value for the observed counts (P=0.0005). **D)** Ct Values and Normalized CM Enrichment of REX and RIX Sequences in the CM fraction. All sequences were cloned under a RNA polymerase III promoter, and their expression in CM was initially normalized against mir-16. Subsequently, the values were further normalized against the corresponding expression of the sequences in IC. REX-1 and REX-5 have

significantly lower Ct's than RIX sequences, and they show significantly higher EV enrichment relative to RIX controls, resulting in a combined Fisher's P values of P<1e-100 and P=1e-4 respectively.

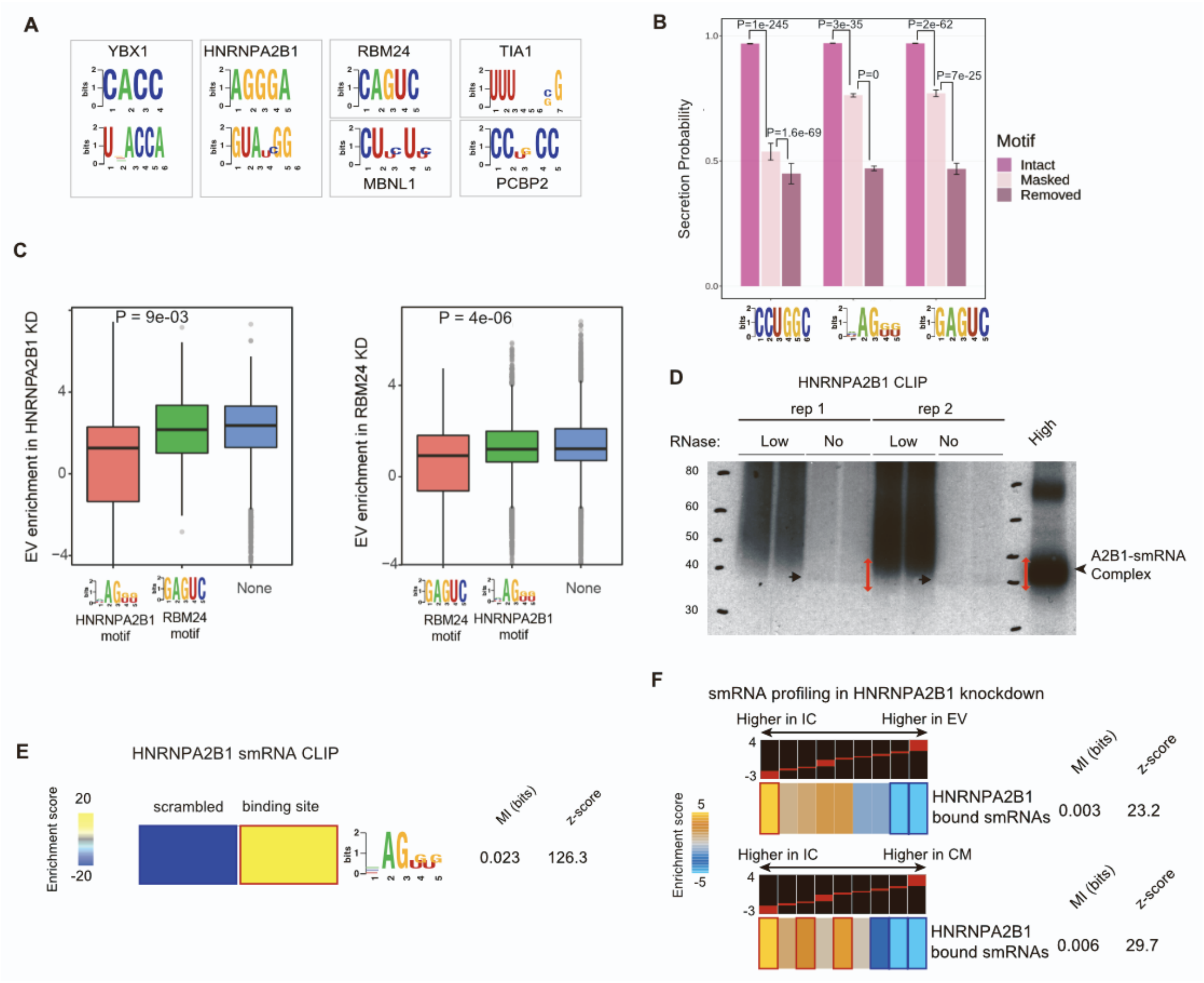

Supplemental Figure 3. ExoGRU RBP motif discovery and CLIPseq to identify small RNA secretion mechanisms, related to figure 3. A) RNA binding protein motifs found by exoGRU to be enriched in ECX sequences and their corresponding RBPs. **B)** Comparative analysis of the model's secretion probability before and after masking (or deleting) enriched RBP motifs in the dataset, including 80 sequences with the CCUGGC motif, 304 sequences with the GAGUC motif, and 1888 sequences with the [ACU]AG[GU][GU] pattern. Each bar in the graph illustrates the average secretion probability before and after the masking or deletion of motifs, with the standard error of the mean (SEM) represented by the error bars atop each bar. Statistical significance was assessed using a Wilcoxon test, comparing the secretion probabilities before and after motif masking. To mask the matched motifs, we set the corresponding inputs to 0 across one-hot encoded channel; to remove the motif, we simply deleted that

portion of the input sequence. **C)** The first graph illustrates the enrichment of small RNAs containing HNRNPA2B1 motifs, RBM24 motifs, or neither in extracellular vesicles isolated from MDA cells with reduced expression of HNRNPA2B1 protein. A notable reduction in the enrichment of small RNAs containing HNRNPA2B1 motifs is observed in comparison to those containing RBM24 motifs (p-value = 9e-03). The second graph depicts a similar analysis but in MDA samples with depleted RBM24 protein. A significant decrease in the enrichment of small RNAs containing RBM24 motifs is observed compared to those containing HNRNPA2B1 motifs (p-value = 4e-06). P-values are calculated using Mann-Whitney U test. The Y axis represents the ratio of EV/IC of these motifs in the KD vs CTRL cell line. **D)** Image of radiolabeled RNA bound to HNRNPA2B1 via CLIP. Protein-RNA complexes were treated with no, low and high RNase after crosslinking. The black arrows point to the faint but visible small RNA band in the no RNAse lane. To retrieve the small RNAs bound by HNRNPA2B1, we excised the membrane in the 40-50kDa range for the no-RNase lanes, which corresponds to the artificially created small RNA-HNRNPA2B1 complex in the high-RNase lane (this range is marked by a double-ended red arrows). **E)** Heatmap illustrates the enrichment of HNRNPA2B1 motifs within HNRNPA2B1 binding sites identified through CLIP-seq, compared to scrambled sequences (with di-nucleotide frequency held constant). Red and bolded borders show statistically significant enrichments, as determined by a hypergeometric test (corrected $P < 0.05$). MI value and associated *z*-score are shown. **F)** Heatmap showing pattern of enrichment or depletion of HNRNPA2B1-bound small RNA sequences (captured by CLIP-seq) in EV and CM fractions upon HNRNPA2B1 knockdown. The panels with black bins show how the sequences are partitioned into equally populated bins based on their EV and CM enrichment measures, going from left (lowly expressed in EV/CM) to right (highly expressed in EV/CM). In the heatmap representation, a gold entry marks the enrichment of the HNRNPA2B1-bound small RNA in its corresponding EV or CM expression bins (measured by log-transformed hypergeometric *P*-values), while a light-blue entry indicates HNRNPA2B1-bound small RNA depletion in the bin. Red and blue borders mark highly significant motif enrichments and depletions, respectively. MI value and associated *z*-score are shown.

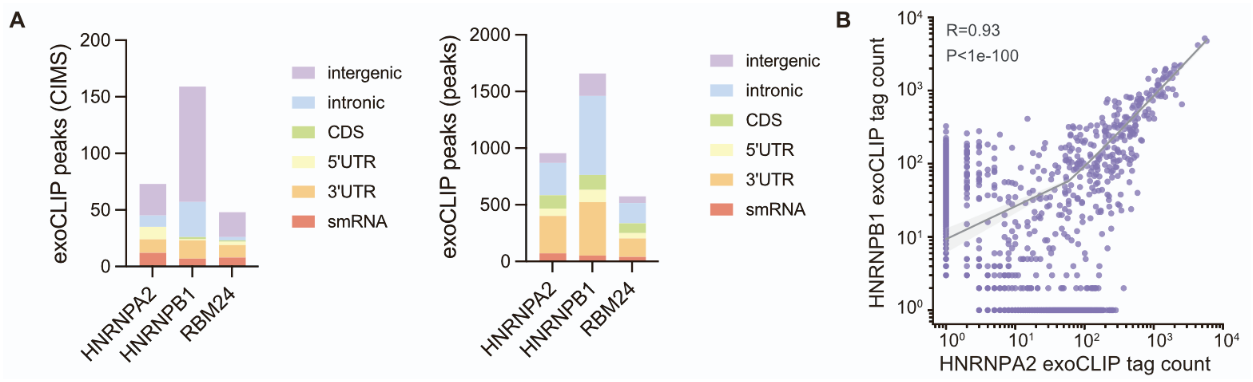

Supplemental Figure 4. Annotated exoCLIP sequencing results, related to figure 4. A) Use of CLIP Toolkit to analyze the HNRNPA2, HNRNPB1 and RBM24 exoCLIP results. For each RNA-binding protein (RBP), two distinct methods were applied to identify peaks. The first plot shows crosslinking induced mutations (CIMs),while the second plot relies on the peak signal or sequence coverage. These plots reveal a substantial abundance of RNA targets associated with each RBP, and these RNA targets were further categorized into various small RNA (smRNA) subtypes.**B)** Scatter plot indicates a high correlation between the distribution of HNRNPA2 and HNRNPB1 tags extracted from exoCLIP data (R=0.93). The plot also shows specific binding sites for these two isoforms.

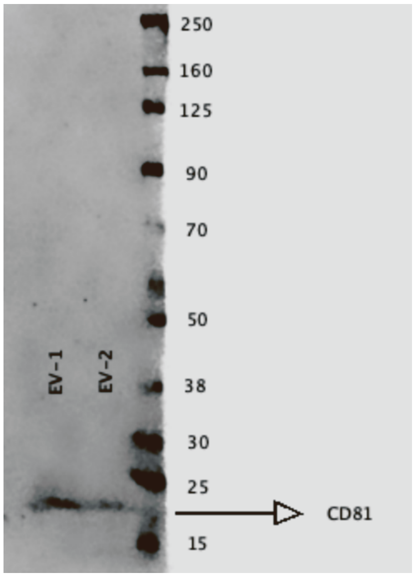

Supplemental Figure 5. Validation of PEG precipitation method in EV isolation, related to the STAR Methods: RNA isolation from conditioned media (CM) and extracellular vesicles (EV). A western blot image illustrating the presence of CD81 protein, a common exosomal marker in EV samples isolated from MDA-MB-231 conditioned media using the PEG precipitation method.
